# Guidance on minimum information requirements (MIR) from designing to reporting human biomonitoring (HBM)

**DOI:** 10.1016/j.envint.2025.109601

**Published:** 2025-06-16

**Authors:** Maryam Zare Jeddi, Karen S. Galea, Jillian Ashley-Martin, Julianne Nassif, Tyler Pollock, Devika Poddalgoda, Konstantinos M. Kasiotis, Kyriaki Machera, Holger M. Koch, Marta Esteban López, Ming Kei Chung, Jihyon Kil, Kate Jones, Adrian Covaci, Yu Ait Bamai, Mariana F. Fernandez, Robert Pasanen Kase, Henriqueta Louro, Maria J. Silva, Tiina Santonen, Andromachi Katsonouri, Argelia Castaño, Lesliam Quirós-Alcalá, Elizabeth Ziying Lin, Krystal Pollitt, Ana Virgolino, Paul T.J. Scheepers, Lisa Jo Melnyk, Vicente Mustieles, Ana Isabel Cañas Portilla, Susana Viegas, Natalie von Goetz, Ovnair Sepai, Emily Bird, Thomas Göen, Silvia Fustinoni, Manosij Ghosh, Hubert Dirven, Jung-Hwan Kwon, Courtney Carignan, Yuki Mizuno, Yuki Ito, Yankai Xia, Shoji F. Nakayama, Konstantinos C. Makris, Patrick J. Parsons, Melissa Gonzales, Michael Bader, Maria Dusinska, Aziza Menouni, Radu Corneliu Duca, Kaoutar Chbihi, Samir El Jaafari, Lode Godderis, An van Nieuwenhuyse, Asif Qureshi, Imran Ali, Carla Costa Trindade, Joao Paulo Teixeira, Alena Bartonova, Giovanna Tranfo, Karine Audouze, Steven Verpaele, Judy LaKind, Hans Mol, Jos Bessems, Barbara Magagna, Maisarah Nasution Waras, Alison Connolly, Marc Nascarella, Wonho Yang, Po-Chin Huang, Jueun Lee, Henri Heussen, Ozlem Goksel, Masud Yunesian, Leo W.Y. Yeung, Gustavo Souza, Ana Maria Vekic, Erin N. Haynes, Nancy B. Hopf

**Affiliations:** a Shell Global Solutions Internationals BV, the Netherlands; b Life Science Intelligence Consulting, the Netherlands; c Institute of Occupational Medicine (IOM), Edinburgh, UK; d Environmental Health Science and Research Bureau, Health Canada, 269 Laurier Ave W, Ottawa, ON K1A 0K9, Canada; e Association of Public Health Laboratories, Bethesda, MD, the United States of America; f Existing Substances Risk Assessment Bureau, Health Canada, 269 Laurier Ave W, Ottawa, ON K1A 0K9, Canada; g Benaki Phytopathological Institute, Laboratory of Pesticides’ Toxicology, 8, Stephanou Delta Street, 14561 Kifissia, Athens, Greece; h Institute for Prevention and Occupational Medicine of the German Social Accident Insurance, Institute of the Ruhr University Bochum (IPA), Germany; i National Center for Environmental Health, Instituto de Salud Carlos III, 28220 Majadahonda, Madrid, Spain; j JC School of Public Health and Primary Care, The Chinese University of Hong Kong, Hong Kong, PR China; k Institute of Environment, Energy and Sustainability, The Chinese University of Hong Kong, Hong Kong, PR China; l Li Ka Shing Institute of Health Sciences, The Chinese University of Hong Kong, Hong Kong, PR China; m Environmental Health Research Division, National Institute of Environmental Research, Ministry of Environment, Incheon 22689, Republic of Korea; n Health and Safety Executive, Buxton SK17 9JN, UK; o Toxicological Center, University of Antwerp, Universiteitsplein 1, 2610 Wilrijk, Belgium; p Center for Environmental and Health Sciences, Hokkaido University, Kita 12, Nishi 7, Kita-ku, Sapporo, Japan; q University of Granada, Spain, Instituto de Investigación Biosanitaria (ibs.GRANADA), Consortium for Biomedical Research in Epidemiology and Public Health (CIBERESP), Spain; r State Secretariat for Economic Affairs SECO, CH, Switzerland; s National Institute of Health Doutor Ricardo Jorge, Department of Human Genetics, Lisbon and ToxOmics - Centre for Toxicogenomics and Human Health, NOVA Medical School, Universidade NOVA de Lisboa, Lisbon, Portugal; t Finnish Institute of Occupational Health, Helsinki, Finland; u State General Laboratory, Ministry of Health, PO Box 28648 Nicosia, Cyprus; v Comprehensive Health Research Centre (CHRC), NOVA Medical School, Universidade NOVA de Lisboa, Portugal; w Department of Environmental Health and Engineering, Johns Hopkins University Bloomberg School of Public Health, Baltimore, MD, the United States of America; x Environmental Health Sciences, Yale School of Public Health, New Haven, CT 06510, the United States of America; y EnviHeB Lab, Instituto de Saúde Ambiental, Faculdade de Medicina da Universidade de Lisboa, Lisboa, Portugal; z Radboud Institute for Biological and Environmental Sciences, Radboud University, Nijmegen, the Netherlands; aa US EPA Office of Research and Development, Center for Public Health and Environmental Assessment, Cincinnati, the United States of America; ab NOVA National School of Public Health, Public Health Research Centre, Comprehensive Health Research Center, CHRC, REAL, CCAL, NOVA University Lisbon, Lisbon, Portugal; ac Federal Office of Public Health, Berne, Switzerland; ad General Toxicology and Biomonitoring, Radiation Chemical Climate and Environment Division, UK Health Security Agency, UK; ae Friedrich-Alexander-Universität Erlangen-Nürnberg, Institute and Outpatient Clinic of Occupational, Social and Environmental Medicine, Erlangen, Germany; af Department of Clinical Sciences and Community Health, University of Milan, Milan, Italy; ag Department of Public Health and Primary Care, Environment and Health Unit, KU Leuven, Leuven, Belgium; ah Dept of Chemical Toxicology, Division of Climate and Environmental Health, Norwegian Institute of Public Health, Oslo, Norway; ai Division of Environmental Science and Ecological Engineering, Korea University, Republic of Korea; aj Department of Food Science and Human Nutrition, Department of Pharmacology and Toxicology, Michigan State University, East Lansing, US, the United States of America; ak Department of Environmental Health Sciences, Mailman School of Public Health, Columbia University, the United States of America; al Department of Occupational and Environmental Health, Nagoya City University Graduate School of Medical Science, Nagoya, Japan; am Key Laboratory of Modern Toxicology of Ministry of Education, School of Public Health, Nanjing Medical University, Nanjing 211166, PR China; an Exposure Dynamics Research Section, Health and Environmental Risk Division, National Institute for Environmental Studies, 16-2 Onogawa, Tsukuba, Ibaraki 305-8506, Japan; ao Cyprus International Institute for Environmental and Public Health, School of Health Sciences, Cyprus University of Technology, Irinis 95, 3041 Limassol, Cyprus; ap Division of Environmental Health Sciences, Wadsworth Center, New York State Department of Health, Albany, NY, the United States of America; aq Department of Environmental Health Sciences, College of Integrated Health Sciences, University at Albany, Albany, NY, the United States of America; ar Tulane School of Public Health and Tropical Medicine - Environmental Health Sciences, Tidewater Building, 1440 Canal Street, #8360, New Orleans, LA 70112, the United States of America; as BASF SE, Corporate Health Management, Ludwigshafen, Germany; at The Health Effects Laboratory, Department of Environmental Chemistry and Health Effects, Climate and Environmental Research Institute NILU, Kjeller, Norway; au Department of Health Protection, Laboratoire National de Santé, 1, Rue Louis Rech, L-3555 Dudelange, Luxembourg; av Human Epidemiology and Environmental Health Research Team, Faculty of Sciences, Moulay Ismail University of Meknes, Meknes 50000, Morocco; aw Department of Climate Change & Department of Civil Engineering, IIT Hyderabad, Sangareddy 502285, India; ax Swedish Chemicals Agency, Health Assessment/Authorisation, Stockholm, Sweden; ay Department of Environmental Health, National Institute of Health, Porto, Portugal and EPIUnit—Instituto de Saúde Pública da Universidade do Porto, Porto, Portugal; az Department of Urban Environment and Industry, Climate and Environmental Research Institute NILU, Kjeller, Norway; ba Italian Institute Against Accidents at Work (INAIL) Department of Occupational and Environmental Medicine, Epidemiology and Hygiene, Italy; bb Université Paris Cité. Inserm U1124, Paris, France; bc NiPERA Inc., 2525 Meridian Parkway, Suite 240, Durham, NC 27713, the United States of America; bd Belgian Center for Occupational Hygiene (BeCOH), Leuven, Belgium; be LaKind Associates, LLC, Department of Epidemiology and Public Health, University of Maryland School of Medicine, the United States of America; bf Wageningen Food Safety Research (WFSR), Wageningen University & Research, Wageningen, the Netherlands; bg VITO Environmental Intelligence, Flemish Institute for Technologcal Research (VITO), Mol, Belgium; bh GO FAIR Foundation, Leiden, NL, the Netherlands; bi Department of Toxicology, Advanced Medical and Dental Institute, Universiti Sains Malaysia, 13200 Kepala Batas, P. Pinang, Malaysia; bj UCD Centre for Safety & Health at Work, School of Public Health, Physiotherapy, and Sports Science, University College Dublin, Dublin, Ireland; bk Massachusetts College of Pharmacy and Health Sciences University, Boston, MA, the United States of America; bl Department of Health & Safety, Daegu Catholic University, South Korea; bm National Institute of Environmental Health Sciences, National Health Research Institutes, Miaoli 35053, Taiwan; bn Stoffenmanager^®^, Van Heuven Goedhartlaan 121, 1181 KK Amstelveen, the Netherlands; bo Ege University, Faculty of Medicine. Laboratory of Occupational/Environmental Respiratory Diseases and Asthma. EgeSAM (Ege University Translational Pulmonary Research Center), 35100 Izmir, Turkiye; bp Department of Environmental Health Engineering, School of Public Health, Tehran University of Medical Sciences, Tehran, Iran; bq Man-Technology-Environment (MTM) Research Centre, School of Science and Technology, Örebro University, 701 82 Örebro, Sweden; br Department of Environmental and Occupational Health Surveillance, Secretariat of Health and Environment Surveillance, Ministry of Health, Brazil; bs Department of Epidemiology and Environmental Health, College of Public Health, University of Kentucky, the United States of America; bt Unisanté, University Center for Primary Care and Public Health & University of Lausanne, Lausanne, Switzerland

**Keywords:** Harmonization, FAIR, Metadata requirements, Contextual information, Chemical monitoring, Exposome

## Abstract

Human biomonitoring (HBM) provides an integrated chemical exposures assessment considering all routes and sources of exposure. The accurate interpretation and comparability of biomarkers of exposure and effect depend on harmonized, quality-assured sampling, processing, and analysis. Currently, the lack of broadly accepted guidance on minimum information required for collecting and reporting HBM data, hinders comparability between studies. Furthermore, it prevents HBM from reaching its full potential as a reliable approach for assessing and managing the risks of human exposure to chemicals.

The European Chapter of the International Society of Exposure Science HBM Working Group (ISES Europe HBM working group) has established a global human biomonitoring community network (HBM Global Network) to develop a guidance to define the minimum information to be collected and reported in HBM, called the “Minimum Information Requirements for Human Biomonitoring (MIR-HBM)”. This work builds on previous efforts to harmonize HBM worldwide.

The MIR-HBM guidance covers all phases of HBM from the design phase to the effective communication of results. By carefully defining MIR for all phases, researchers and health professionals can make their HBM studies and programs are robust, reproducible, and meaningful. Acceptance and implementation of MIR-HBM Guidelines in both the general population and occupational fields would improve the interpretability and regulatory utility of HBM data. While implementation challenges remain—such as varying local capacities, and ethical and legal differences at the national levels, this initiative represents an important step toward harmonizing HBM practice and supports an ongoing dialogue among policymakers, legal experts, and scientists to effectively address these challenges. Leveraging the data and insights from HBM, policymakers can develop more effective strategies to protect public health and ensure safer working environments.

## 1. Introduction

Human exposure to chemicals found in the environment (e.g., air, water, soil), food, and consumer products as well as during work-related activities may result in adverse effects on human health (CDC 2018b; Shoeb et al. 2023). Exposure assessment plays a crucial role in the identification, evaluation, and control of health risks, both in occupationally exposed and general populations. Human biomonitoring (HBM) is defined as the measurement of chemical substances and/or their metabolites, or markers of body’s response to the external factors, in human biological matrices such as tissues, cells or body fluids (e.g., blood, urine, maternal milk, etc.) (Heinemeyer et al. 2022; Knopp 1995; Louro et al. 2019). HBM is a robust approach in epidemiology, environmental health surveillance, occupational health and human health risk assessments, providing critical data that aid in detecting and evaluating chemical exposure levels and assessing alterations in biological endpoints or adverse health effects resulting from such exposures (Desye 2022; EFSA et al. 2024; Morgenstern and Thomas 1993; NRC 2006). Thus, HBM assesses the internal exposure to various chemicals from all routes (e.g., oral, dermal, inhalation) and sources, leading to a direct and more precise assessment of the internal exposure concentrations (Cascio et al. 2022; de Jong et al. 2022; Ladeira 2024; Ladeira and Viegas 2016; Louro et al. 2019; Luijten et al. 2023; Poddalgoda et al. 2024; Viegas et al. 2024; WHO 2023). HBM data may also be used in a probabilistic manner for human risk assessment to support regulations, by comparing exposure biomarker concentrations (parent compound or associated metabolites concentration in a given biological matrix) to specific human biomonitoring guidance values (Aylward et al. 2013; de Jong et al. 2022; Faure et al. 2020; Ganzleben et al. 2017; Luijten et al. 2023; Nakayama et al. 2023; Ruiz et al. 2014; Ruiz et al. 2010; Satarug et al. 2013; St-Amand et al. 2014; Ubong et al. 2023). Some of these values can be found in the HB2GVs dashboard. Combining HBM data with additional information concerning the primary sources, routes of exposure and temporal variability, along with physiologically-based (bio)kinetic (PBK) modelling, adverse outcome pathways (AOPs) and new approach methodologies (NAMs) can enhance our understanding of exposure dynamics and their related health implications (Angerer et al. 2011; Arnold et al. 2013; Goksel et al. 2024; Ladeira 2024; Marx-Stoelting et al. 2023; O’Flaherty 2000; Ruiz et al. 2014; Ubong et al. 2023).

The recognition of the benefits offered by HBM in environmental and occupational health has catalyzed a significant growth in the number of HBM studies (Ladeira and Viegas 2016; Ubong et al. 2023; Zare Jeddi et al. 2022). European initiatives like the Consortium to Perform Human Biomonitoring on a European Scale (COPHES), the European Human Biomonitoring Initiative (HBM4EU), the EU-funded Partnership for the Assessment of Risk from Chemicals (PARC) and The Environmental Exposure Assessment Research Infrastructure (EIRENE) have emphasized the need for biomonitoring studies (Kolossa-Gehring et al. 2023; Marx-Stoelting et al. 2023; Schindler et al. 2014). However, the lack of harmonization, which involves making data from different sources compatible and comparable, limits the potential of HBM (Siontorou and Batzias 2011). This variability complicates the comparison, integration, and aggregation of data across different sources or studies, ultimately minimizing the influence these studies can have on regulatory frameworks and other policy-related issues. Substantial efforts are necessary to identify, collect, curate, and harmonize results from multiple HBM studies to construct databases of sufficient quality that can be used for instance for producing summary information, knowledge graphs, developing exposure modeling tools, systematic reviews and *meta*-analyses or conducting risk assessments (Zare Jeddi et al. 2023; Zare Jeddi et al. 2021b). The high quality HBM databases should contain both well-defined metadata and contextual information. Metadata refers to the descriptive information that provides context and details about the data collected. Metadata are crucial for data management, replication of studies, and data sharing among researchers and reduce any ambiguity. Contextual data refer to additional information that may affect exposure and consequently, the interpretation of results.

Although several guidelines and recommendations are available (see Supplementary Material Annex A), most existing guidelines address how to conduct biomonitoring using various elements of “good practices”, but do not define mandatory minimum information that must be captured, placing less emphasis on the alignment and harmonization of data collection and documentation of metadata. Moreover, many stakholders may not be aware of these resources or know how to utilize them effectively. Diverse frameworks across continents and the considerable complexity of many protocols further complicate the issue. To date, there is no specific minimum information requirement (MIR) guidance for data generated in HBM (examples of existing MIRs in other files provided in Annex B). Therefore, it is imperative to establish minimum information requirements for collecting and reporting HBM data to ensure the generation of more reliable, harmonizable, accessible, reusable, manageable and interpretable data. HBM can be a study, or a program (HBM programs in general population such as NHANES and HBM4EU or occupational HBM programs at company level) and we refer to either of them as “HBM”. The MIR guidance fills this gap in HBM by offering an actionable template supported by a broad consortium of international experts from academia, government, and industry, under the HBM Global Network.

The European chapter of the International Society of Exposure Science, HBM working group (ISES Europe HBM working group) has established a community known as the HBM Global Network. This network comprises an international group of researchers and analysts from 27 countries, including 38 representatives from academia, 19 from governmental organizations, and 7 from the industrial sector—the authors of this publication— to define the minimum information that must be collected and reported in HBM studies. Herein we propose a guidance document for “Minimum Information Requirements for HBM” (MIR-HBM).

## 2. Minimum information requirements for HBM (MIR-HBM)

The MIR-HBM is a list of criteria/parameters to support a useful conceptualization, description, planning and interpretation of human biomonitoring (HBM) for exposure assessment. MIR-HBM enhances data quality, harmonization, accessibility, and interpretability and enables a thorough understanding of the HBM data obtained. A successful HBM study or program design relies on the development and execution of a well-organized plan, which includes: a primary question, selection criteria, ethical considerations and approval, participant recruitment and enrollment, biological sample and data collection, management and storage of samples, sample preparation, harmonized or comparable chemical analyses, data management and statistical analysis, and reporting of results (Fiddicke et al. 2021). For each of these steps, MIR should be defined to ensure sufficient quality/reliability and relevance of the generated data. Additionally, a successful HBM design and well-organized plan should incorporate a post-evaluation phase. This phase allows for a retrospective analysis of the HBM’s successes and shortcomings, facilitating the identification of best practices and lessons learned to inform future endeavors. These aspects will be embedded in the www.FAIREHR.com (Findable, Accessible, Interoperable, and Reusable Environmental and Health Registry) platform.

The main desirable outcomes of implementing MIR-HBM include:

1. **Improved HBM design and reporting:** The purpose of the HBM defines the design. The MIR-HBM guidance helps to transparently report the HBM purpose, and the level of rigor used in the design, execution, and analysis. This is crucial for generating high-quality data and maximizing its potential for reuse.
2. **Enhanced harmonization, comparability, and interpretability**: The collection of sufficient contextual data enhances the interpretability of HBM results. The contextual information gathered plays a critical role in identifying possible sources and determinants of the observed exposure as HBM provides information from all routes and sources of exposure. Even for identified or presumed exposure sources, details such as exposure duration, frequency, and, in the case of occupational exposure, specific determinants (e.g., risk management measures) enhance the understanding of exposure pathways and risk factors. Establishment of MIR-HBM ensures that the type and level of detail in data collected from HBM are harmonized and comparable. This is crucial for conducting *meta*-analyses and for comparing data across different populations and time periods.
3. **Improved QA/QC:** MIR-HBM supports quality management systems (QMS) in HBM by ensuring that quality assurance and quality control (QA/QC) procedures and protocols are used to confirm that the methodologies are comparable, reliable, and valid. Making all components of the HBM process available is crucial for transparency, reproducibility, and reusability.
4. **Facilitated interconnectivity and free public accessibility of data:** MIR-HBM will enhance the integration of established HBM initiatives with governmental databases (e.g., the Organization for Economic Co-operation and Development (OECD) eChemPortal, and the European Common Data Platform on Chemicals (EC 2023)) ensuring the long-term sustainability and accessibility of HBM findings for regulatory purposes and public health intervention aimed at exposure reduction. The MIR-HBM aligns with the FAIR (Findable, Accessible, Interoperable, and Reusable) data principles, enhancing the accessibility, comprehension, exchange, and reuse of HBM outputs.

## 3. Structure of MIR-HBM

**Fig. 1.**
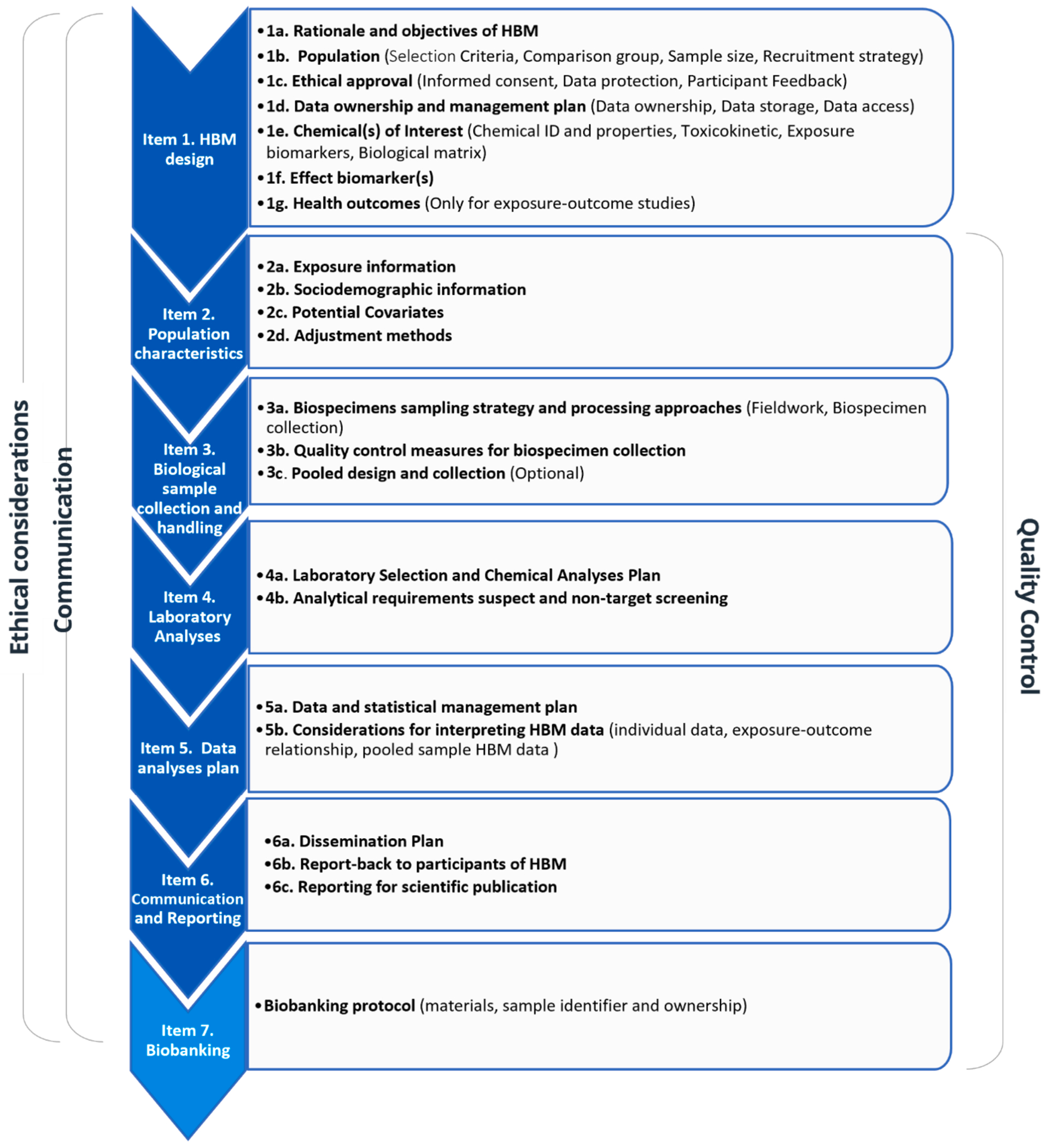
General Workflow of Human Biomonitoring (HBM) with the relevant Minimum information requirements (MIR) topics depicted on the right side for each step.

Once HBM is considered as an appropriate method for assessing exposure to a given chemical, the design and structure of the HBM study or program can be developed accordingly as outlined here in the MIR-HBM guidance. The HBM Global Network identified the key steps and components of HBM, from initial planning to final reporting, to develop six essential and one recommended MIR-HBM criteria (Fig. 1) (Fiddicke et al. 2021).

Although beyond the scope of these guidance, we recognize the importance of engaging the community (e.g. stakeholders) throughout the HBM process and communicating the outputs. Those impacted by the potential exposure can provide valuable input at each step, from HBM design and planning to data report-back. However, adherence to defined MIR is non-negotiable (Haines et al. 2011; Israel et al. 2005; O’Fallon and Dearry 2002).

### Item 1. HBM design

Setting a clear definition of rationale, goals, and questions is fundamental in conducting HBM. The purpose of the HBM defines the design of a study or program.

The minimum information required for this section includes the following:

#### 1a. Rationale and objectives of HBM

- The rationale and context of HBM should be described including defining the primary question, background information and identification of sub questions to address the main question. Objectives are the specific, measurable goals therefore, evidence underpinning these objectives should be outlined, explaining why the selected approach is best suited to answer the question and how it relates to similar HBM initiatives. This should include relevant references to previous work.

#### 1b. Population

The population may consist of the general population, or any sub-population defined, e.g., by age, sex, or employment in the case of occupational studies. Participants can be randomly selected to obtain a sample representing a specific or a general population, e.g., for the purpose of describing background exposure. If not randomly selected, criteria for recruitment shall be defined and applied.

- Selection criteria: inclusion and exclusion criteria for participants, (e.g. sex, age range, health status, urban or rural environment, occupation, or others) should be defined according to study objectives and the research questions. For certain study designs and populations, such as volunteer studies, intervention studies and in-company occupational biomonitoring, the design may be intentionally non-representative for convenience or to capture only the intended individuals. For studies with representative designs, the study population can be compared to census population characteristics to ensure comparability or to identify and clearly describe significant differences.
- Comparison group: where appropriate, the biomonitoring results of an exposed group can be compared with the results from an internal or external (e.g., nearby city with similar characteristics but background exposure) reference group. In occupational HBM, production workers with a specified exposure can be, e.g., compared with workers in the same plant with no known – or minimal – exposure such as administrative workers. In other cases, a nearby non-exposed population with similar characteristics may be used. The HBM results can also be compared to existing data from more generic population (such as from the National Health and Nutrition Examination Survey (NHANES) in the U.S., the Canadian Health Measures Survey (CHMS) in Canada, and the German Environmental Survey (GerES) in Germany). Especially when measuring effect biomarkers or assessing a health outcome, participants in exposed and control groups should be matched, e.g. by age, lifestyle such as smoking or other confounders or effect-modifiers. It is also important to realize that other contaminants may cause similar effects and that an effect biomarker may help assessing exposure to chemical mixtures while they can be less or non-specific for the single contaminant.
- Sample size determination: Sample size should be clearly defined and justified by power calculation according to the study design or biomonitoring program using well-defined statistical parameters. The target number of participants should ensure that the HBM is sufficiently powered (Althubaiti 2023; CDC and Environmental Public Health Tracking Program 2023; Giroux 2007; Tolonen et al. 2022a; Tolonen et al. 2022b). Achieving sufficient power can sometimes be challenging for understudied, hard-to-reach, and small populations, which often experience significant health disparities. Therefore, obtaining relevant and accurate HBM metadata and contextual information for these groups, even if underpowered, is indispensable for informing public health programs and decision-making. Overall, inclusion and exclusion criteria as well as loss to follow-up, false positives/negatives, detection rates, integrity of samples should be documented and made available to identify attrition and exclusions.
- Recruitment strategy: outline how participants (source population and target population) will be identified, contacted, and enrolled in the HBM study or program (where and when). Make a detailed plan with the place where the HBM will take place and a timeline for recruitment (whether seasonal or throughout the year). Ensure that participants in the comparison group are recruited during the same time of the year as the exposed participants, etc. The ethics committee of the local or institutional authority should provide approvals for all relevant study parts and recruitment cannot start before these approvals have been provided.

#### 1c. Ethical approval

HBM studies should follow ethical principles set up in legal and ethical guidance documents such as ethical considerations, informed consent, confidentiality, data handling, and the protection of participants in HBM research,^1,2^ (Erden and Brey 2021; Kapp 2013; Knudsen et al. 2023; Manno et al. 2014).

- General Informed consent: Participants must be fully informed about the study’s purpose, procedures, potential risks and benefits, data protection measures, what is expected of them, how results will be shared (individually or in aggregate), who will have access to the data, and the study’s funding source. They must also be informed of their right to withdraw from the study at any time without penalty. Written informed consent must be obtained; for minors, consent must be acknowledged and/or signed by a parent or legal guardian. This should be done in a manner that minimizes the potential for coercion or undue influence, particularly if there is monetary incentive for participating. It must be ensured that participants’ personal and health information is kept confidential and that adequate safeguards are in place to protect privacy. The Ethics committee accredited under national law or Institutional Review Board approval depending on the intent and scope of the study (surveillance, community investigation or applied research) is required (Council for International Organizations of Medical Sciences 2023; Linda 2022; Shrestha and Dunn 2019; WMA 2014).

Additional informed consent includes:

- **Informed consent for biobanking** of the biological samples and for anticipated future analyses (might be needed following the country or institutional rules / legislation) (Colcelli et al. 2023; Slokenberga et al. 2021).
- **Informed consent for data sharing** purposes (as needed and as possible) and data linking with administrative, health, and other datasets (might be needed following the country or institutional rules / legislation).
- **Informed consent for sample sharing**, locally or abroad, within a Material Transfer Agreement (MTA) (might be needed following the country or institutional rules/ legislation). Additionally, a Data Transfer Agreement might be needed in some cases.
- Data protection and confidentiality: As with all studies involving human participants, the study needs to be in accordance with the Declaration of Helsinki. Personal and sensitive data collected from human must be securely stored and protected against unauthorized access. Where possible, data should be anonymized to prevent the identification of individual participants (Pseudo-anonymization or confidentiality of the identifiable data)(Ghosh et al. 2023; Govarts et al. 2022; Martorana et al. 2022). Access to personal data should be restricted to authorized personnel directly involved in the study or program and especially in the data analysis. Compliance with local and national laws, and with regulatory standards is required. In the U.S., it is common to obtain a Certificate of Confidentiality from the National Institute of Health (NIH), which protects against forced disclosure of personal identifying information. In Europe, compliance with the EU Regulation 2016/6 on the General Data Protection Regulation (GDPR) is essential (GDPR 2016). In other parts of the world, researchers need to refer to the guidelines of the national commission or authority for the protection of personal data. Researchers must ensure that all personal data is handled in accordance with these regulations to avoid legal repercussions and maintain the integrity of the study.
- Social justice and risk- benefit analysis: HBM studies should ensure equitable treatment of all participants, considering vulnerable populations, and the benefits of research are shared fairly. HBM studies must weigh the potential benefits of the research (e.g., public health improvements) against the potential risks to participants (e.g., stigmatization, etc.).
- Participant Feedback: It should be outlined in which manner participants will receive feedback and what mechanisms will be put in place to address any distress that may be caused by the HBM results. Additionally, a request from the participant to not receive information or be contacted (as in the right not to know) should be respected.

#### 1d. Data ownership and management plan

Making raw data available to enable a thorough evaluation of outcomes and conclusions is important. Therefore, having a data ownership and management plan in place is needed to facilitate access to raw data for reuse. The plan should outline the responsibilities and protocols for handling and safeguarding data throughout the research process in line with FAIR principles (EC 2016). In practice, obtaining raw data for risk assessment purposes is often challenging. Consequently, risk assessors must rely on the processed data published by the authors, which can have significant implications. This reliance may even result in the decision to exclude human data from risk assessments.

- Ownership of the data: It should be clearly defined who holds ownership of the data collected during the HBM (e.g., institution, research team, funding agency, or company). The ownership should also clarify rights related to data sharing, publication, and use in future studies. Additionally, it should be reported how third parties can access the (raw) data.
- Data storage for HBM studies: in publications, details on secure methods for data storage and sharing of anonymized or pseudonymized data should be given. This should include the physical and digital storage solutions that will be used to protect the integrity and confidentiality of the data in line with FAIR principles. Data should be stored in encrypted, secure databases with regular backups to prevent loss, such as the Information Platform for Chemical Monitoring (IPCHEM), and European Common Data platform.
- Data storage for in-company HBM programs: Data storage and access depends on the company data infrastructure. It is recommended to store anonymized or pseudonymized data for exposure assessment and risk management purposes. For medical surveillance, individual data can be stored according to the medical protocols and employees should receive a copy of history of monitoring data upon request at end of employment (change of job, or retirement).
- Data access: Access to the raw data should be restricted based on roles and responsibilities. Clear protocols for data access must be in place to regulate how data is shared within the research team, a company and external stakeholders, including the use of data transfer agreements and secure file transfer methods. In research, the publication should also specify how and when the data will be made publicly available, adhering to ethical guidelines, data-sharing policies, and any relevant legal or regulatory frameworks.

#### 1e. Chemical(s) of interest

This section is divided into four parts which describe the chemical(s) identity, toxicokinetic information, exposure biomarkers and biological matrices.

- Chemical identifiers (ID) and properties: Identification of chemicals should include at least one of the chemical ID approaches including the use of technical or IUPAC name, unique formula identifier (UFI), valid CAS-number, InChi, and/or Simplified Molecular Input Line Entry System (SMILES) and should provide the composition of product formulation/mixtures, when needed. When necessary, include relevant physico-chemical parameters such as log P_o/w_, pK_a_, vapor pressures.
- Toxicokinetics (TK): The rate of Absorption, Distribution, Metabolism, and Elimination (ADME) are important to understand the relationship between occurrence of exposure and the measured concentration of a chemical and its metabolites in the body (Reale et al. 2024). This information can be obtained from controlled human exposure studies or if these do not exist, from animal (Arnold et al. 2013) or NAMs studies (Hoogstraaten et al. 2024). It is important to identify the most prominent and specific biomarkers to monitor systematic availability. Additionally, urinary excretion fraction is essential to extrapolate from internal values (represented by urine concentrations) to external exposure estimates (Nehring et al. 2020; Ringbeck et al. 2021; Schettgen et al. 2021; Wrobel et al. 2023). The choice of the appropriate biological matrix, in which the parent compound or its metabolites is measured also depends on the kinetic properties of the substance within the body (Aylward et al. 2014) (see below section on Biological matrix and Temporality). There are also new approaches where in silico or in vitro models are used to predict TK in humans, such as the high-throughput toxicokinetic (HTTK) from US EPA (Breen et al. 2021) and TKPlate from the European Food Safety Authority (EFSA) (EFSA et al. 2023).
- Exposure biomarkers: Exposure biomarkers (either parent compounds and/or metabolites) reflect the human internal exposure to a specific chemical agent (Heinemeyer et al. 2022) and in case of chemicals with short half-lives, present a time-variable concentration profile that is associated with temporal patterns of exposure and elimination kinetics (Ladeira and Viegas, 2016). Exceptions are made for chemicals that are persistent in the environment, bioaccumulate in people and/or wildlife, and are toxic (called PBTs) (Ladeira and Viegas, 2016).
  - **Bioavailability and relevance:** When selecting suitable exposure biomarkers, it is imperative to determine whether the studied chemical has sufficient bioavailability, ensuring that it can be effectively absorbed and measured within the body. Beside physiochemical properties of chemicals, bioavailability can be influenced by numerous parameters such as route of exposure, human physiological characteristics (intrinsic factors such as genetics, lifestage, pregnancy, gender), and extrinsic factors (e.g., diet, medication, medical conditions, climate) (Ladeira and Viegas, 2016; LaKind et al., 2014). There should be clear evidence provided for the selection and justification of the proposed exposure biomarker (s) and the respective matrix (biomarker/matrix combination) based on the objective of the study (Vorkamp et al., 2021). If the exposure biomarker is monitored for risk assessment purposes, even substances with minimum bioavailability may be used in various risk assessment approaches, such as the low exposure approach as explained in Poddalgoda et al. (2024) and Health-Canada (2016). The MIR-HBM can also be used for research focused on biomarker discovery including determining biomarker’s bioavailability and relevance.
  - **Specificity:** Another important factor is understanding whether the biomarker (metabolite) of exposure is derived from the substance of interest or is formed by endogen processes in the body (LaKind et al. 2014). If the metabolite is formed from multiple parent compounds (i.e. when multiple chemicals have common metabolites), then this metabolite is not a specific biomarker for the substance of interest. If such a biomarker is used, it needs to be interpreted with caution (Calafat et al. 2015; Hays et al. 2008; Macey et al. 2025; Poddalgoda et al. 2024; Zidek et al. 2017).
  - **Sensitivity:** The selection of biomarkers should consider the prevalence of chemicals at trace levels and the ability to detect them in the biological matrix (NRC 2006). Therefore, the prerequisites include analytical methods able to measure the substance or metabolites at sufficient sensitivity (APHL 2019).
- Temporality and Biological matrix: The kinetic parameters, such as elimination half-life dictate not only the biomonitoring matrix, but also the timing of the biological sample collection (Reale et al. 2024; Vorkamp et al. 2021). In occupational HBM, the sampling time should be aligned with exposure and elimination half-life of the investigated substance (Hopf et al. 2025; Hopf et al. 2024; OECD 2022). When the timing of the exposure is not known (unknown temporality) in general population, the biological sample collection depends mainly on the half-life of the substance. The rationale behind the actual biomarker-matrix combination used should be clearly addressed when different possibilities are available. The biomarker-matrix combination is also linked to existing HBM guidance values as outlined in their documentations (Angerer et al. 2011; Apel et al. 2020; Macey et al. 2025; Nakayama et al. 2023; Ruiz et al. 2014; Ruiz et al. 2010; Satarug et al. 2013; Vorkamp et al. 2021; Weistenhöfer et al. 2023).

#### 1f. Effect biomarker(s) (for exposure assessment of chemical mixture)

- Selection of effect biomarkers: Effect biomarkers focus on the body’s response to external factors, while clinical biomarkers are primarily used for diagnosing and managing diseases. Effect biomarkers are currently the most direct way to assess the impact of both known and unknown exposures, especially to mixtures of chemicals with the same mode of action. A wide variety of both available and promising new biomarkers can be selected for effect biomonitoring. The OECD Working Parties of Hazard and Exposure Assessment group recently developed a conventional framework on effect biomarkers (Zare Jeddi et al. 2021a) and an international expert group is currently working on guiding principles on the potential of using certain effect biomarkers in human health risk assessment due to exposures to chemical mixtures. A finalization of this OECD activity is planned for 2025 and will provide occupational biomonitoring effect levels for several relevant effect biomarkers. For these types of biomarkers, the rationale for the selection of the suitable biomarker(s) in the proposed matrix and health outcome should be provided.

#### 1g. Health outcomes (Only for exposure-outcome studies)

##### Health outcome(s) of interest:

When assessing the association between exposure to chemicals and health outcomes, it is essential to collect detailed information on the type/definition of a health outcome, method of measurements and diagnosis details (including self-reported outcomes, doctor-diagnosed outcomes (based on assessments in individual studies or via outcome registers) and diagnostic/medical test results). Where possible, standardized assessment criteria (self-report or direct measurement) should be used to capture information about health outcomes.

### Item 2. Population characteristics

This section specifies contextual information needed to be able to interpret the biomarker outcomes.

#### 2a. Exposure information

The OECD Harmonised Template (OHT) 301 can be utilized to collect this information in a standardized manner (OECD 2024) for occupational HBM. However, it is more difficult to establish patterns of exposure for the general population since a variety of factors impact exposure and lead to greater variability in exposure (Arnold et al. 2013). Potential sources of exposure can often be determined using questionnaires regarding environmental-, sociodemographic-, and lifestyle factors. For instance, study setting (residence location) is widely used in environmental epidemiology (Stürmer and Brookhart 2013). All these questionnaires will be accommodated on the www.FAIREHR.com platform for ease of use and accessibility.

#### 2b. Sociodemographic characteristics

Sociodemographic characteristics refers to data that describes the combination of social and demographic factors of individuals or populations (Vo et al. 2023). Sociodemographic characteristics help in understanding the context in which people live, work, and interact, providing insights into patterns, trends, and disparities in various aspects of life, including health, education, and socioeconomic status (SES). These might impact exposure levels and consequently health outcomes. Sociodemographic information is essential for analyzing social trends, identifying at-risk populations, and developing policies that address specific community needs. Examples of sociodemographic and anthropometric (body measurement) information are presented in Annex C. The available questionnaires for collecting sociodemographic information are listed in the www.FAIREHR.com platform.

#### 2c. Potential covariates

Covariates are variables that are possibly predictive of the outcome and are important to consider when assessing the effect of an exposure on health outcomes. Appropriate covariates should be selected based on their potential impact on the relationship between the exposure and the health outcome based on existing evidence. Additionally, confounding factors and effect modifiers should be accounted for. Confounding factors are associated with both the exposure and the outcome and can distort or bias the observed relationship between them. Effect modifiers are variables that modify the strength or direction of the association between an exposure and an outcome. Only data that is necessary for the interpretation of HBM results should be collected. In research, collecting information on potential confounders requires ethical approval, as this data may be classified as personally identifiable information (PII). In statistical models, covariates are included to control for their potential effects, ensuring the relationship between the independent variable (exposure, treatment, or predictor) and the dependent variable (outcome) is not confounded or biased by other factors. Examples of the most common potential covariates (listed in Annex C Supplementary material) and questionnaires developed under the HBM4EU project are available in Annex D Table S3. In the upcoming years, new questionnaires are expected from the PARC project as well. All these questionnaires will be accommodated on the www.FAIREHR.com platform for ease of use and accessibility.

#### 2d. Adjustment methods

In HBM, adjustment of raw measurement data accounts for individual variability and ensure accurate comparisons within populations. Adjustment methods correct for variability in factors like hydration, metabolism, or body size that could affect the measured concentration of chemicals. For instance, adjustments for creatinine and specific gravity for urine volume are often used to account for variations in hydration status and voiding volume (Aylward et al. 2014; Kuiper et al. 2022; Weaver et al. 2016). (Table S1 Annex D supplementary material) shows a list of commonly used adjustment methods. Transparent reporting of the specific method used for adjustment is essential for ensuring consistency and reproducibility across studies. Reporting both uncorrected raw measurements alongside adjusted data is recommended to ensure future reuse of datasets, for example for deriving HBM guidance values.

### Item 3. Biological specimens (biospecimens) collection and handling

Effective biomonitoring requires careful planning and execution of sample collection, storage, and transportation to ensure the integrity and reliability of the data obtained (Calafat and Needham 2009; CDC 2018a). Several bodies and organizations such as OECD (OECD 2022), the Association of Public Health Laboratories (APHL) (APHL 2019), the International Agency for Research on Cancer (IARC) (IARC 2016), the Centers for Disease Control and Prevention (CDC) (CDC 2018a), the National Institutes of Health (NIH) (NIH 2023) and the German Epidemiological Society (Hoffmann et al. 2019), have published guidelines and recommendations for sampling, processing, transportation, and storage of biological samples that should be contemplated throughout the HBM. Supplementary material, Annex D Table S4 indicates some relevant standard operating procedures (SOPs) developed under HBM4EU initiative, available in open access. In the upcoming years, new SOPs are expected from the European project PARC as well. All SOPs will be implemented in the www.FAIREHR.com platform.

#### 3a. Biological specimens (biospecimens) sampling strategy and processing approaches

Strict protocols for sample collection, including the timing of sampling, handling, transport, and storage, are particularly important when monitoring exposure to chemicals that are ubiquitous environmental contaminants or when exposure can occur in different settings, through various sources, or when dealing with unstable chemicals or environmental degradants (Calafat and Needham 2009). The sampling approach, including the sample volume and method of sampling, may vary between adults and non-adults. Different types of biospecimen might require specific handling conditions to prevent degradation and cross-contamination.

- Fieldwork Manual**:** Before starting the fieldwork, it is recommended that the analytical laboratory conducting the analyses is consulted to determine SOPs for specimen collection, transportation and storage (CDC 2018a). Participant identification, coding, informed consent to be signed, type of sample required (collecting the correct biospecimen component), container type, sample labeling, and special handling and storage should be determined before the fieldwork starts. A checklist with the necessary consumables to carry out sampling and transportation of samples should be compiled, pre-checked for completeness, and used in the field by scientific personnel.

Field work manual should also include description on how to control for contaminations, proper materials for collection and storage, and how to accommodate analyte stability as a part of Quality Assurance (QA) measures for field sampling.

- Sample (Biospecimen) collection: Biological sample (biospecimen) collection can be conducted using various methods, depending on the specific biomarker-matrix combination. This includes both self-sampling by participants (for urine, saliva, stool, breast milk samples) and sampling by clinical professionals (blood samples). Chain-of-custody procedures are a necessary element to ensure the quality and utility of the samples from the time samples are collected until sample disposal (ASTM D4840-99(2018)e1). Table 1 outlines minimum information required for biological sample collection.

#### 3b. Quality control measures for biospecimen collection

Samples should be contamination-free from time of collection to time of measurement (e.g., by use of certified analyte-free collection supplies and reference materials, and appropriate use of blanks both in the field and lab). Sample processing procedures, examination of potential external contamination, and assessment of the accuracy and precision of the measurement can be evaluated by using three common methods including field blanks, sample splits and blind quality controls (QCs) (CDC 2018a).

- **Field blanks** are sample matrices (e.g., urine, blood, saliva, stool, breast milk) with no analyte present following the whole route from sample collection, processing, and storage before analysis to assess potential contamination (Calafat 2016). It is important and valuable to maintain a collection instrument repository or library of empty storage vials. Without field blanks, it is not possible to ascertain whether sample contamination is occurring, which would result in an upward bias in concentration by an unknown amount (LaKind et al. 2019). When targeting ubiquitous chemicals, particular attention is required to ensure that no cross-contamination occurs.
- **Samples split** in the field undergo the same pre-analytical and analytical treatment thus they can be used to assess the quality of aliquoting technique and the precision of the analytical measurement.
- **Blind QCs** are the same matrix (e.g., urine, blood, saliva, stool, breast milk) that are generally added to the analytical run by the field staff or the laboratory and are subjected to the same analytical processing as the collected human samples. Blind QCs are particularly useful for identifying sample mix- ups or errors in the sequence of samples.

#### 3c. Pooled design and collection (optional)

In societies with limited resources, analyzing individual samples may not be affordable. Furthermore, some chemicals may exist in low concentrations in body fluids and need a large volume of media (such as serum) to be detectable in samples. In these cases, a composite (pooled) sample may be desirable. In this method, a combination of samples drawn from multiple individuals with the same characteristics (such as the same sex, age group, or geographic area) will be analyzed as “one sample” and the results will be generalized to the same group of individuals (UNEP 2017). Pooling samples results in the dilution of the investigated biomarker value (Bader et al. 2020). For instance, if a biomarker is present in one sample and this sample is pooled with four others that do not contain the biomarker, the concentration is diluted fivefold. Consequently, more sensitive detection methods with lower limits of detection (LODs) may be required to identify the biomarker. As such, pooled samples may not require participant consent if the individual samples are de-identified before pooling and results do not need to be communicated to the individual participants (Heffernan et al. 2014). The pooling design must be carefully planned and implemented. Only those variables that are considered as part of the pooling design (e. g., sex, age group, or region) can be stratified as part of the subsequent data analyses (cannot stratify by other variables not considered in the pooling design (e.g., socioeconomic status, race/ethnicity, or product use)).

### Item 4. Laboratory analyses

Hight quality laboratory analysis is needed to produce reliable, comparable (i.e., reproducible) and timely analytical results. Unreliable or erroneous laboratory data that are used in the context of HBM findings can result in incorrect interpretation and conclusions. The chemical analytical method must be developed or exist before proceeding with sample collection. A robust quality management system (QMS) supports the accuracy of the analytical measurements, investigates and prevents quality failures and ensures a successful HBM study or program (Esteban-López et al. 2021; Kannan et al. 2021).

#### 4a. Laboratory Selection and Chemical Analyses Plan

Appropriate analytical methods with sufficient working range and LOD/LOQ that are fit-for-purpose are needed for determining exposure biomarkers at trace levels (Magnusson and Örnemark 2014). Accreditation status should be considered when selecting a suitable laboratory (e.g., ISO CEI 17025). Occupational HBM should follow the required standards in occupational exposure assessment (ISO 20581). Furthermore, the laboratory should maintain participation in internationally recognizing External Quality Assessment Schemes (EQAS).

Following multifaceted quality assurance protocols among laboratories has been demonstrated to ensure reliable and reproducible data for several classes of organic chemicals (Kannan et al. 2021). Harmonized framework within laboratories such as consortium of laboratories established under the Children’s Health Exposure Analysis Resource (CHEAR) should account for instrumental performance, matrix effects, and inherent procedural limitations. Traceability and harmonization are closely linked (Esteban-López et al. 2015; Esteban-López et al. 2021; Schindler et al. 2014). In multicenter studies when each laboratory establishes a traceability chain, typically using calibration standards and independent verifications can be made by use of Standard Reference Materials^®^ (SRMs), certified reference materials (CRMs), in-house quality control material (QCM), or matrix-matched standards (Galusha et al. 2021).

Key laboratory metadata is explained in Table 2.

#### 4b. Analytical requirements for suspect and non-target screening

In quantitative target analysis, analytical reference standards are available and used for method validation and quality control. This is not possible in case of suspect and non-target screening. By definition, samples are analyzed without reference standards, or the analytes are even completely unknown. The performance of the analytical method (extraction/ cleanup/instrumental measurement) is typically monitored using QC-mix(es) of compounds (ranging from 5 to 100 compounds). In suspect screening, the compounds should represent the physical chemical properties of the suspect compound list. For a comprehensive non-target analysis, the mixture should encompass a diverse range of chemicals. This approach ensures that the analysis can effectively cover the entire chemical space, allowing for the detection of a broad spectrum of chemicals present in the sample. For the compounds included in the mixture, QC can be performed in a similar way as described for the quantitative target analysis (see 4a) to gain insight in measurement reproducibility and background artifacts (Caballero-Casero et al. 2021; Monteiro Bastos da Silva et al. 2021; Newton et al.; Vitale et al. 2022).

### Item 5. Data analyses plan

#### 5a. Data and statistical management plan

When defining the statistical techniques to be used for data analysis, several key aspects must be addressed and recorded to ensure a robust and accurate evaluation of the data as shown in Table 3.

#### 5b. Considerations for interpretation of HBM results

Interpretation of the HBM results depends on the question and availability of guidance values. The availability of national population-level HBM data (reference values-RVs) is one of the key requirements of conducting HBM-based risk assessments for the general population (Poddalgoda et al. 2024).

##### Interpretation of individual HBM data:

- **Comparing the results with population reference values (RVs):** When assessing the overall exposure of the studied sample population, HBM data or biomarker concentrations may be compared with other populations or to reference concentrations from national surveys or large population studies, when the study designs are comparable, and the populations can be compared by age. HBM data from such surveys or studies constitute a relevant source to determine RVs which are based on the upper end of the exposure distribution, such as 95th percentile concentrations. Population RVs are particularly relevant in the absence of HBM guidance values, either when such values are not available or cannot be derived (Nakayama et al. 2023).
- **Comparing the results with HBM guidance values**: Guidance values are biomarker levels established to assess whether the detected levels of a chemical in human biological samples are of concern. These values vary by country, organization, chemical and are derived from human or animal data using uncertainty factors or modelling approaches, such as PBK modelling (Apel et al. 2020; Macey et al. 2025; Nakayama et al. 2023). Different type of HBM guidance values are listed in (Table S2 Annex D supplementary material). If HBM guidance values are not available for a chemical, other guidance values (e.g., Acceptable Daily Intake values (ADI)) can also be used, when risk assessors can make sound assumptions of potential exposure and have sufficient human metabolism data. To compare with ADI, the systemic concentration (internal concentration) should be converted to an external exposure estimate using either kinetic data or regression analysis. These comparisons may have limitations, e.g. ADIs were derived for uptake by ingestion. This should be considered when comparisons are made for populations with (additional) uptake by other routes of exposure such as inhalation or dermal absorption.

##### Interpretation of exposure-outcome relationship:

When the HBM objective is to evaluate exposure-outcome relationships, several considerations need to be taken into account including the linear or non-linear relationship between exposure biomarker levels and health outcome, specificity and sensitivity of the outcome being studied, confounding factors, statistical modeling techniques (e.g., Forward Dosimetry), casualty assessment, and uncertainties (Clewell et al., 2008).

When comparing HBM data from different studies (Inter-country comparisons) following factors can impact the interpretation of the results:

- Methodological differences (e.g., assignment of values for measurements below the limit of detection, use of field blanks and analytical approaches).
- Adjustment methods used.
- Population characteristics (e.g., age, body weight and fasting): Any chemical with a strong dietary component for exposure and short physiological half-life could be impacted by differences in fasting times. Conversion of concentration to intake is useful for several purposes including comparisons with guidance values but requires incorporation of body weight to convert units of concentration (e.g., ng/mL) to intake (e.g., ng/kg/day). Thus, any inter-country differences in body weight should be addressed by comparisons of intake rather than concentration (LaKind et al. 2019).
- While the interpretation of individual data for personal use is not addressed here, it remains an important issue for communication.

##### Considerations for interpreting the pooled sample HBM data

HBM data obtained from pooled samples provides a good estimate of how much of a chemical is present in a defined population or sub-population. When interpreting results from pooled samples the following should be considered (HealthCanada 2020):

- The absence of a chemical in a pooled sample does not necessarily rule out exposure of the relevant population to that chemical. In fact, if one or a few individual samples in the pooled sample have measurable levels of the chemical, but other individual samples in the pool have no or trace levels of the chemical, it is possible that the overall concentration in the pooled sample will be non-detectable. Therefore, for pooled sample analyses, only very high sensitivity analytical methods should be used.
- By using pooled samples, you may not be able to examine the relation between exposure to chemical(s) of interest and health effects, because health research and health risk assessment usually requires substantial information about individuals to properly conduct (e.g., medication use, chronic conditions, time of diagnosis).
- Only the arithmetic mean can be reported for chemicals measured in pooled samples. The geometric mean cannot be reliably generated for pooled samples, since the act of pooling the samples distorts the distribution. Importantly, the arithmetic mean of pooled sample data cannot be compared with geometric means reported for other HBM studies using individual samples.
- Generally, it is not possible to reliably calculate percentiles in the population or estimate variance within the population when using pooled samples. This may restrict the ability to conduct hypothesis testing of pooled data, such as monitoring trends over time, assessing determinants of exposure, and epidemiological studies. The extent to which this work is restricted depends on the nature of the pooling design – pooling more samples into fewer pools is generally more restrictive than pooling fewer samples into more pools.

### Item 6. Communication and reporting

#### 6a. Dissemination

Development of a methodology and strategy for communicating results to stakeholders is imperative (Korfmacher and Brody, 2023). First, it should identify all the target audiences for the HBM results communication, such as policymakers and regulatory agencies, researchers and scientific community, industry associations, NGOs, public health officials, the public or workers. The plan should also define the key messages or data to be communicated, such as health risks, policy recommendations, or new scientific discoveries. Next, it must specify the timeline for sharing the findings at various stages, including preliminary results, final reports, and potential follow-up updates. The plan should remain flexible, allowing for updates as the project progresses to ensure timely and effective communication. Additionally, it is essential to determine the channels for communication, such as report back to participants (see item 6b), peer-reviewed publications, infographics, webinars, conferences, press releases, websites, or social media, ensuring that the message is tailored to the intended audience. In the communication plan (Pseudo)-anonymization or confidentiality of the data should be addressed. The evaluation of the reach and impact of the reporting process, as well as the broader impact of the project itself, should be conducted on a regular basis to ensure continuous improvement in the dissemination of results. For instance, the LIFE GoPro for project offers a Layman’s Report (as requested by the European Commission) summarizing the objectives, assumptions, and results in a concise and accessible format. The key point is ensuring that information is delivered in a language and format that is appropriate for the target audience to make scientific findings easily understandable to the general public (LAYMAN’s Report, 2021).

#### 6b. Report-back to participants of HBM

##### General population:

Consent forms should include an opt-in or opt-out statement for the reporting back of individual and/or overall results. Personalized, context-rich feedback should be the norm, ensuring that participants receive relevant and meaningful information about the HBM’s findings. A template for participant report-back is sometimes requested by the ethical committee to be submitted to obtain approval for the communication of HBM findings to participants. The MIR-HBM template for communication (Table 4) provides a consistent and transparent framework for reporting back personal HBM data, ensuring that participants receive clear, accurate, and meaningful information. This template can be modified according to the questions and requirements of the study and the population. The MIR-HBM template promotes ethical transparency in HBM research, improves participant engagement, and empowers individuals with their own exposure data, fostering a better understanding of personal and public health risks.

##### Worker population

When assessing the exposure under a certain scenario at the workplace, the same template as for the general population (Table 4) can be used for participating workers. However, the participant should be advised to inform their occupational medical doctor, unless it has been agreed in advance that the results will be directly communicated via their occupational medical doctor. In addition, the overall summary of the results at group level is advisable to be communicated to the company while preserving the confidentiality of individual data (Viegas et al. 2020).

#### 6c. Reporting for scientific publication

HBM is an exposure assessment tool when using exposure biomarkers. However, HBM can also be used to relate exposure to early biochemical changes in the body using effect biomarkers, resembling a typical environmental epidemiology study. To date, there is no specific checklist for reporting of HBM in scientific publications. The Strengthening the Reporting of Observational Studies in Epidemiology (STROBE) Initiative developed a set of recommendations, including a checklist of 22 items, outlining what should be included in an accurate and complete report of an observational study. This checklist can be used when reporting HBM using effect biomarkers. The HBM Global Network has updated and modified the existing STROBE checklist to better align to MIR-HBM for reporting of HBM for exposure assessment. The checklist called “MIR-HBM checklist”, is presented in Table 5. The MIR-HBM checklist will be digitalized in www.FAIREHR.com platform and reviewers can use this for peer review purposes.

### Item 7_Recommended_. Biobanking (materials, sample identifier and ownership)

Irrespective of the specific biological samples being collected in HBM—acknowledging that not all studies will gather the full range of specimens—careful consideration must be given to the biobanking of unused samples or of samples conserved after analysis. This includes defining their potential fate, future uses, and limitations, establishing clear protocols for obtaining informed consent from participants for long term storage of samples or their usage in future research projects or for purposes other than the ones described at the moment of sample collection, and determining the allowable duration for sample storage, with particular attention to the stability of the samples during long-term storage. Additionally, there is a crucial need for long-term storage and maintenance resources and for record keeping on the history of samples.

#### 7a. Biobanking protocol.

Nowadays, with the rapid advancements in the effect biomarker identification and utilization, it is essential to have a complete biobanking protocol. It is imperative that biobanks include a wide array of biological specimens such as serum, plasma, whole blood, red blood cells, and white blood cells as a minimum requirement. Given the post-COVID-19 trend towards remote participation in research studies, there is also value in collecting and storing biological specimens (e.g. toenails (Gutiérrez-González et al. 2019) that can be easily collected at home, shipped, and stored. Comprehensive biobanking allows for a broader range of analyses and better supports the development of new insights. RNA preservation would also be advisable. The long-term storage and maintenance of biobanks requires notable personnel, financial, and structural resources; it is, therefore, necessary to outline the associated costs and feasibility of biobank creation at the outset of sample collection.

- Ownership: It is critical to clearly define the ownership of biological samples stored in biobanks and to establish the terms of use agreements when external researchers request access to these samples or associated results for secondary studies. Such agreements must specify the conditions under which the samples can be utilized, ensuring that the original study’s objectives are respected, and participant confidentiality is maintained. These agreements should also address data-sharing responsibilities, intellectual property rights, and ethical considerations, including re-consent if the intended future research deviates significantly from the information stated in the original consent provided by participants. The consent process must clearly explain to participants how their data and biological samples will be stored, managed, and potentially used in future studies.
- Compliance with legal and regulatory frameworks regarding sample biobanking: considerations related to the destruction of samples once they are no longer viable or required for future research should be stated. Establishing transparent governance over the biobanking process is essential to maintain ethical integrity and scientific rigor in the use and management of these valuable biological resources. Biobanking is usually subject to ethics approval.

### Next steps

**Fig. 2.**
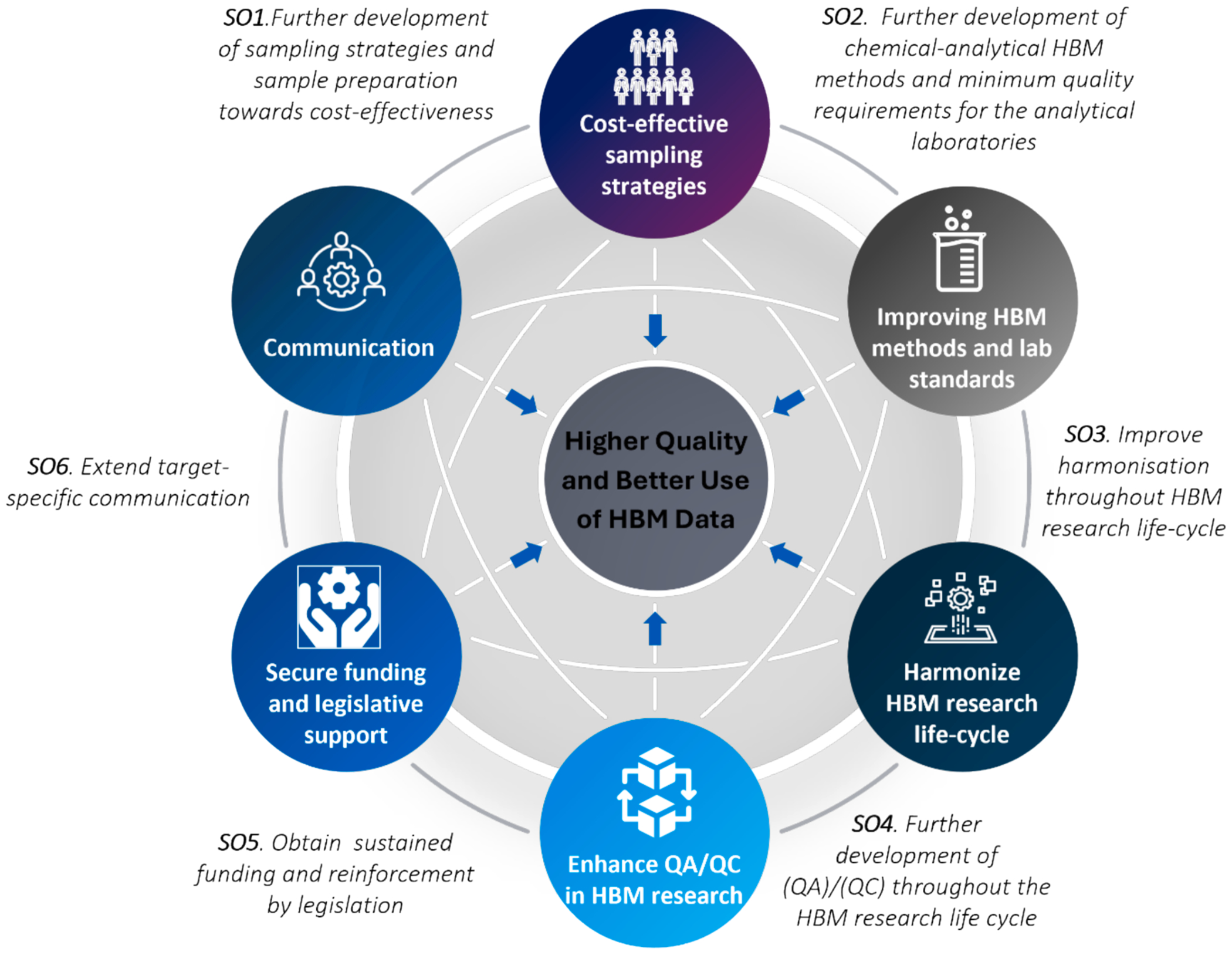
The strategic objectives (SO) for human biomonitoring (HBM) are to generate high-quality data and to utilize it to its full potential.

In line with the need for more and high quality HBM data (Calafat 2012), the ISES Europe HBM Working Group has outlined six strategic objectives advocating for advancement and sustained use of HBM (Zare Jeddi et al. 2022). The development of MIR-HBM and its implementation in the www.FAIREHR.com platform are actions taken by this working group and the HBM Global Network to fulfill Strategic Objective 1: further developing sampling strategies and sample preparation towards cost-effectiveness, and Strategic Objective 2: improving sample preparation and harmonization throughout the HBM research life cycle (Fig. 2). The MIR-HBM will be translated to human and machine-readable questions and integrated in the global registry platform, www.FAIREHR.com, as envisioned in Zare Jeddi et al. (2023). The primary objective of this registry platform is to ensure that HBM metadata is available in a timely manner, even before the study planning phase begins. The comprehensive nature of MIR-HBM and its implementation in the www.FAIREHR.com platform offers distinct advantages without imposing an excessive burden on researchers. Here, we tried to include as much relevant information as possible to aid interpretation and reuse of HBM data. Depending on the study type and objectives, not all elements might be considered as mandatory. The current MIR-HBM guidance is tailored for exposure biomarkers as these are complementary to the exposure assessment, which is related to a more preventive strategy. However, given the increasing use of effect biomarkers in the assessment of exposure to chemical mixtures, we envisage the development of a complementary MIR framework focused on effect biomarkers. This would support the harmonized integration of exposure and effect data in human health risk assessments. In addition, other effect biomarker activities are ongoing with the focus to derive regulatory actionable mixture thresholds for effect biomarkers according to the principles of the Adverse Outcome Pathway.

Adherence to MIR supports the reliability and reusability of the data but also enables the scientific community to build upon previous work, fostering a collaborative environment conducive to the advancement of environmental health science. It is important that the MIR-HBM is deployed and that data exchange formats are further developed by institutes and organizations that endorse the use of HBM, such as CDC National Health and Nutrition Examination Survey (NHANES). NHANES provides comprehensive view of the chemical exposure landscape in the US population over time and help identify trends for individual chemicals and various population groups in the format of Biomonitoring Data Tables for Environmental Chemicals (Stanfield et al. 2024). The HBM Global Network is committed to promoting the use of this guidance to foster wider acceptance and integration of HBM harmonization. This effort aims to ultimately improve the overall quality and reliability of HBM data. This will also enable better application of artificial intelligence (AI) in HBM research, reducing the burden on data generators and allowing them to focus more on critical analysis and interpretation of HBM data.

Overall, www.FAIREHR.com aims to align with the ethical principles outlined in the World Medical Association’s Declaration of Helsinki 2013 states in article 35:

“*Every research study involving human subjects must be registered in a publicly accessible database before recruitment of the first subject*”.

## 4. Conclusion

Human biomonitoring is a powerful tool for assessing internal chemical exposures in both general and occupational populations. Its ability to measure the total exposure across multiple pathways and sources makes it uniquely valuable for risk assessment regarding the exposure to hazardous substances. However, the full potential of HBM has not yet been realized due to persistent challenges, including inconsistent metadata documentation, lack of harmonized protocols, and limited comparability between studies.

This MIR-HBM guidance addresses these gaps by providing a structured, globally applicable framework for study design, conduct and reporting. The strengths of this approach lie in its ability to increase data quality, reproducibility, and reusability while aligning with FAIR principles and facilitating regulatory acceptance. Through well-defined metadata categories, MIR-HBM supports transparency, improves interpretation, and allows more robust integration of HBM data across contexts and populations. Nevertheless, limitations remain. These include varying degrees of feasibility in different resource settings, the need for ethical and legal harmonization of consent and data protection practices. Issues around strengths and weaknesses of HBM studies as such, and around integrating HBM in regulatory risk assessment and policy frameworks have been discussed in our previous publication Zare Jeddi et al. (2022) (strategy paper). Additionally, further efforts are needed to develop a complementary guidance for biomarkers of effect.

By promoting the widespread adoption of MIR-HBM, we aim to increase the impact of HBM on science, policy, and health decision-making, ensuring that the data generated today can support the public health needs of tomorrow.

## Supplementary Material

Supplement1

## Figures and Tables

**Table 1:** Template for biological sample (biospecimen) metadata collection.

Category	Details	Explanation
Unique Identifier	[Enter participant ID]	A unique code or identifier for each sample to ensure traceability.
Date and Time of biospecimens	[Enter date and time of sample collection correctly]	Date and time of sampling should be recorded consistently to avoid confusion or misreading.
Time of sampling relative to exposure events	[Record the time interval between the chemical exposure and the biospecimen collection	Knowing the right time to collect samples is crucial for accurately detecting and measuring biomarkers. Occupational exposure assessment requires sampling time to correspond to time of exposure (e.g. calculated based on urinary elimination half-life). If samples are taken too early or too late after exposure event, the biomarkers might not be present or detectable. For the general population the time between exposure and sample collection might not be easy to calculate. In general, population-level biomonitoring studies include large samples size to compensate such limitations. Another important step would be using a questionnaire to estimate possible time of exposure. For urinary biomarkers, collecting 24 h urine sample is a suitable solution when the sample size is small. Other factors might influence effect biomarkers and therefore should be collected (such as fasting status, time since last meal, time since last visiting the toilet).
Time between sample collection and pre-analytical sample preparation	[Record time elapsed between sample collection and any pre-analytical sample preparation]	The contextual information regarding time elapsed between sample collection and any preparation procedure helps to accurately interpret exposure levels, considering relevant factors influencing the integrity of the collected samples.
Pre-analytical sample preparation	[Enter method Details]	A sample preparation may be needed in the field (e.g., centrifugation, aliquoting) before cooling/freezing and packaging for transportation to the laboratory for long-term storage and analysis.
Biomarker stability	[Document stability data]	Biomarker stability refers to the ability of a biomarker to remain unchanged and reliable over time under various conditions.
Storage and transport conditions at the sampling point	[Describe storage conditions]	If the sample cannot be delivered to the laboratory immediately, storage condition and time should be followed according to SOP provided by the laboratory, or the World Health Organization (WHO) has published the Laboratory Quality Stepwise Implementation (LQSI) tool in compliance with ISO 15189. This includes conditions/temperature, and any preservatives added during initial storage directly after sampling, local intermediate storage before shipment to the analytical laboratory, and shipment conditions.
Sample contamination	[Describe the QC method]	Include documentation of the steps taken to provide the necessary assurance that the study data is reliable. See section on “Quality control (QC) measures for sampling”
Sample transfer agreements	[Provide sample transfer agreement and chain of custody]	Sample transfer agreements should be established, allowing the provision of clear instructions on the chain of custody. An example has been developed under HBM4EU.

**Table 2:** Template for laboratory metadata checklist – Chemical Analysis Plan for HBM using exposure biomarkers.

Category	Details	Explanation
**Sample Information**		
Sample ID	[Enter Sample ID]	Should be unique and indelible on sample containers/tubes
Sample Type	[Enter Sample Type (e.g., plasma, serum, whole blood, urine, saliva, stool, breast milk)]	
Sampling materials/containers preservatives	[Enter materials used]	Especially for ubiquitous chemicals, sampling materials (e.g. vacutainers with reagents) and containers/tubes for storage may be a source of the target chemical(s). Registration of all materials, including any preservatives, aids in the tracking of potential sources and artifacts. Ideally, field blanks should be included in the sample collection using the same materials but a simulant matrix (e.g. water) instead of the sample to monitor this.
Sample Collection Date	[Enter Collection Date]	For urine samples it is useful to ask the participant to report the date and time of the previous toilet visit as a proxy to the time a biomarker was accumulating in the bladder. From the current and previous time useful parameters can be calculated such midpoint time of urine sample collection and sometimes it is possible to express urinary excretions in units of time (e.g. for use in reverse dosimetry PBK modelling or for comparison to results of published kinetic parameters
Laboratory Storage Conditions	[Enter Storage Conditions]	This includes storage conditions at the analytical laboratory, and whether samples were repetitively thawed/frozen or not.
**Analytes of Interest**	[List Analytes]	Analytes (parent compound and metabolites) should include at least one of the chemical ID approaches including the use of technical or IUPAC name, unique formula identifier (UFI), valid CAS-number, InChi, and/or Simplified Molecular Input Line Entry System (SMILES).
**Analytical Methods**		
Method Used	[Enter technique used, (e.g., GC–MS/MS, LC-MS/MS, LC-HRMS, ICP-MS, ICP-OES)]	A full description of sample preparation (deconjugation if applicable, extraction, cleanup), instrumental analysis, internal and/or external calibration/quantification, standard addition, and QCs included must be made available. If the method has been published and is used as such, then reference to the publication suffices. Otherwise, the description or ideally SOP should be documented as part of the study dossier.
Method performance characteristics	[Enter description of validation protocol]	Methods used must have been validated before sample analysis. In HBM there is no general harmonized validation protocol, but at national level (e.g. (M. Bader et al. 2010) or in related domains (forensic analysis, medicines) guidance is provided (EMA 2024). Scientific Working Group for Forensic Toxicology (SWGTOX) Standard Practices for Method Validation in Forensic Toxicology published a pivotal document on bioanalytical method validation (SWGTOX 2013).
	[Enter trueness]	Trueness expressed as bias, ideally based on CRM (certified reference material) or RM (reference material) or otherwise by spiking of the biomarker to the sample.
	[Enter precision]	Precision, both repeatability (intra-assay) and intermediate precision (inter-assay) expressed as relative standard deviation (RSD_r_, RSD_i_). Precision, expressed as the coefficient of variation (% CV), should not exceed 20 % within and between runs at each concentration level (low, medium, and high) as a minimum requirement.
Procedures used for calculating or estimating LOD	[Enter method Details, Enter LOD of the analytical instrument, Enter LOD of the analytical method]	Various definitions and procedures exist for calculating or estimating LOD (Gries W et al. 2024). Therefore it is essential to report the definition used and way it was determined.
Limit of Quantification (LOQ)	[Enter LOQ]	It is useful to explain how the LOQ was determined for which type of sample (solvent standard or matrix-mixed standard) and what criteria were used to set the value, e.g. precision limits such as coefficient of variation). If it is a solvent standard it is called the instrumental LOQ, that is used as the method LOQ that is needed to test fit for purpose.
**Quality Assurance and Quality Control (QA/QC)**		
Method validation and accreditation	[Enter validation and accreditation status]	As indicated above, before application the method must have been validated and performance characteristics checked against pre-set criteria that are fit for the purpose of the study.For analytical methods, the relevant accreditation is ISO17025. Here it is relevant that not only the laboratory as such is accredited, but that also the method (chemicals/matrix) itself falls within the scope of the accreditation.
Storage stability	[Enter storage stability data]	In HBM samples are often stored for a longer period of time before analysis (e.g. in biobanking). The stability of analyte in the matrix over time under the applied storage conditions should be investigated and reported. If stability was not established in advance, matrix-mixed standards could be stored alongside the batch of samples to monitor any degradation over time (see below).
Batch control	[Enter positive and negative controls included in each analytical batch]	Each analytical batch (set of samples analyzed in one set together) should include negative and positive controls. Negative controls include (field blanks, procedural blanks, blank samples if available) to identify background artifacts. Positive controls are ideally certified reference materials (CRMs), otherwise spiked samples. These are used to continuously monitor method performance (e.g. Shewhart charts) and provide on-going information on average trueness intermediate precision. Trackers with temperature and humidity sensors can be stored with sample batches during transportation for logging temperature fluctuations over time.
QC: independent performance verification	[Enter participation in PT and outcome]	Laboratories should participate in Proficiency Testing (External quality assurance, EQA) programs or, if not available, other means of interlaboratory comparisons (ILCs) to assess their performance against independently derived assigned values or reference values.
Historical control data	[Enter positive and negative controls data for historical controls]	Laboratories should have a register of control data obtained in previous studies that are useful to assess their performance, particularly, concerning effect biomarkers analysis.
**Data Management**		
Raw machine reading values	[Enter raw data]	Raw data of the instrumental measurement values are increasingly being used as alternatives to statistical methods for handling non-detects, offering the advantage of being free from the distributional assumptions that statistical methods impose. However, these raw values can be challenging to interpret, particularly when zero or negative values are present (Ashley-Martin et al. 2023b; Buckley et al. 2022). Many laboratories establish reporting limits which are “policy decision” that reflects the lab’s confidence in the concentration being reported) and those should be accompanied by raw data.
Lab reporting limits	[A “policy decision” that reflects the lab’s confidence in the concentration being reported]	It is important to agree how/what laboratories will report, whether they should include values above the LOD or only above the LOQ, and establish clear definitions and identification requirements.
Data Storage Location	[Enter Data Storage Location]	Often required as part of the study protocol for ethics approval.
Data Backup Procedures	[Describe Data Backup Procedures]	Laboratoiries should have back up procedures in place.
Data availability	[Explain how data can be accessed]	All reported data by the laboratory should be open, ideally in a standardized format following FAIR data principles.

**Table 3:** Template for statical analyses and data management.

Category	Details	Explanation
Final number of Sample size	[Enter number]	The total number of data points that are included in the data analysis after any necessary data cleaning or filtering should be reported. Reporting rates of non-response (invited participants that do not participate in the study) and loss to follow-up (for longitudinal cohort studies) can provide insight into possible bias.
Methods for handling missing data	[Describe methods used]	This may involve techniques such as multiple imputation or maximum likelihood estimation, depending on the nature of the missing data (random, systematic, etc.).
Methods for handling outliers	[Describe methods used]	Whether outliers are the result of measurement error or true data points, strategies such as robust statistical methods or sensitivity analyses should be employed to mitigate their impact on the overall analysis.
Methods for handling values below the LOD or LOQ	[Describe methods used]	Various approaches are used to handle left-censored values (Chung et al. 2024). These range from model-free substitution by determined values, such as LOD/2, LOD/√2, and MDL/2 (to account for any possible sample matrix effect during sample treatment or analysis), to regression-based approaches such as quantile regression imputation of left-censored data (QRILC) and more advanced Gibbs sampler-based missing value imputation method (Wei and Wang 2021).
Selection of potential covariates	[Describe covariates]	Selection of certain covariates included in the statistical model should be justified based on prior knowledge, literature, or exploratory data analysis.
Methods for handling missing covariates	[Describe methods used]	Methods such as multiple imputation or sensitivity analysis can be applied to minimize bias and retain as much of the dataset as possible. This ensures that the statistical models used are not compromised by incomplete data.
Selection of potential confounders (only for studies addressing health effects)	[Describe confounders]	Directed acyclic graphs are increasingly recognized as an epidemiological tool for identifying the minimum sufficient set of potential confounders necessary to include in etiological analyses (Lederer et al. 2019; Lipsky and Greenland 2022; Williams et al. 2018). Statistical techniques, such as propensity score matching or regression models, can help to adjust for these confounders.
Assessment of normality	[Describe methods used]	Methods for analysis of not normally distributed data. Transformation makes datasets comparable by setting them to a common scale or reference point. Examples of transformation methods include log transformation for skewed databases (extreme exposure values), or percentile rank to assess relative exposure within population.
Descriptive analysis	[Describe statistical methods used]	A statistical method used to summarize and describe the main features of a dataset should be defined. It involves calculating measures such as mean, median, mode, standard deviation, percentiles, and range, providing insights into the distribution, central tendency, and variability of the data. This analysis helps identify exposure trends, establish reference values, and assess the potential health risks related to environmental or occupational exposure to harmful substances (Ashley-Martin et al. 2023a; Lesko et al. 2022).
Statistical models	[Describe statistical models used]	Statistical models selected should align with the research objectives and the type of data being analyzed. For instance, linear regression, logistic regression, or generalized linear models may be suitable depending on the outcome variables. In case there is a hierarchical (multilevel) structure in the data with data that are not independent the use of mixed models is recommended. The underlying distribution of the data must be assessed to determine the appropriateness of these models. In cases where data deviate from a normal distribution, log transformations to reduce skewness and stabilize variance or non-parametric methods may be necessary.
Methods to correct for measurement errors	[Describe methods used]	In instances where there is potential for measurement error, particularly in studies involving nonpersistent chemicals, methods to correct for measurement errors, such as regression calibration or simulation extrapolation, should be outlined to improve the reliability of the results.
Unit of measurement	[Report units]	Unit of measurement for each variable must be clearly defined.
Matrix adjustment (Adjustments to measurements)	[Describe methods used]	Processes and techniques used to modify raw data from biological samples to account for various factors that can influence the results (e.g. normalization).
Sensitivity analyses	[Describe methods used]	Where appropriate, sensitivity analysis should be used to assess the robustness of the results, ensuring that the findings remain consistent across various assumptions and scenarios. This process involves testing the impact of different methodological choices, such as handling missing data, adjusting for confounders, or using alternative statistical models. By performing these analyses, potential biases or uncertainties in the primary analysis can be identified, and the stability of the conclusions under different conditions can be evaluated. Sensitivity analyses are particularly important when there is uncertainty regarding the assumptions or limitations of the data or methods used.
Addressing Inter and intra variability	[Describe methods used]	There are factors that might influence inter- and intra-individual variation in biomarker concentrations leading to two types of variability, interindividual and intraindividual variability in biomonitoring datasets. In cases where repeated observations are available statistical methods should address the dependency of two or more biomonitoring results collected within the same individual. A common example is comparison of biomarker levels in pre-shift spot urine samples collected before the start of worker exposure with a post-shift urine sample collected during a break or after the end of the shift. This is often done to verify that exposure is work-related. These two samples are dependent because they relate to the same study participant that can be used as its own reference. A paired statistical analysis will result in a powered analysis because in this way the analysis will take into account any known and unknown sources of variability.

**Table 4:** The MIR-HBM template for communicating HBM results to study participant (Report back).

Research question-Objective of HBM	Description of the question and objective of the HBM in a clear and layman language.
HBM results (exposure biomarkers levels)	A summary of the individual’s HBM results, when available, and/or a summary of the overall study results, including the levels of specific chemicals or biomarkers detected and how these compare to the study population, and/or available reference or guideline values (e.g., national averages or health-based guidelines targeted for public health screening). Sometimes guidance values are available but usually a comparison is only valid on group level. Link to the full study report, if available.
Contextual Information	Clear explanations of what the detected levels mean, exposure sources, and risk factors. This includes definitions of scientific terms, such as “limit of detection (LOD)” or “exceeding a reference level.”
Personalized Risk Information	Information tailored to the participant’s specific results, explaining potential sources of exposure, and potential health risks based on their biomarker levels, if available. If health risk information is not available or cannot be interpreted for an individual, state this (e.g., The health risk associated with this level of exposure is unknown). Be aware of possible mixture effects when communicating results.
Actionable Recommendations	Practical advice on how to avoid or reduce exposure to harmful substances (so-called ‘no regret measures’), where applicable (e.g., prevention at work, lifestyle, habits or smoking). This might include behavioral, environmental, or policy-level recommendations.
Contact person	Name and contact details of the person who can help to interpret the results and is able to give additional information on the meaning of results (e.g., the study lead). For any questions related to the individual’s health situation, referral to a qualified and authorized healthcare professional (with access to the individual’s medical information) could be arranged.
Links to additional Resources	These include links to trusted sources where participants can learn more about the chemicals they were exposed to, potential health effects, and general recommendations on maintaining health and safety.

**Table 5:** Strengthening the Reporting of HBM for scientific publication: MIR-HBM checklist.

Section/Topic	Item No.	Checklist item
**Title and abstract**		
Identification as Human biomonitoring (HBM)		
Provide an accurate summary of the research design, objectives, methods, principal findings, and study conclusions.		
**Introduction**		
Background and objectives	1a	Include sufficient scientific background to understand the rationale and context for HBM. Clearly describe rationale and objective for HBM. State the research question, and where appropriate, specific hypotheses being tested.
**Methods- Protocol of the study**		
Participants	1b	Give the eligibility criteria, and the sources and methods of selection of participants. Describe the setting, locations, and relevant dates, including recruitment plan, exposure, follow-up (if needed), and timing of sample collection. Report Initial sampling size and statistical power. Important changes to methods. When needed, describe methods of follow-up and rate of attrition.
	1b	When using effect biomarkers additional requirements are needed. For matched studies, give matching criteria and number of exposed and unexposed (Comparison group size and selection criteria).
HBM design	1c	Ethical considerations and Ethical committee approval
	1d	Data ownership and management plan
	1e	Present key elements of HBM design (including chemicals of interest, toxicokinetic, exposure biomarkers, biological matrix)
	1f	Types of biomarkers, the rationale for the selection of the suitable biomarker(s) in the proposed matrix
	1g	When assessing associations between chemical exposures and a health outcome, collect detailed information on the type/definition of a health outcome, method of measurements and diagnosis details and criteria.
Population characteristics	2a, 2b, 2c, 2d	Clearly define methods for collection of all relevant information (e.g., Questionnaires), on exposure, sociodemographic and potential covariates, confounders, and effect modifiers (when needed). For each variable of interest, give sources of data and details of methods of assessment (measurement).
Biological specimen collection	3a, 3b, 3c,	Use the biological sample collection template (Table 1)
Analytical methods	4a, 4b	Use the laboratory metadata template (Table 2)
Statistical method	5a, 5b	Use the statistical analysis check list template (Table 3)
**Results**		
Dissemination plan	6a	Report planned communication to different audiences regarding the HBM results.
Report back to participants	6b	Mention what kind of reporting back to participants is envisioned and practiced to what extent (See the template in Table 4)
**Scientific publication of the HBM results**	6c	
Recruitment		Dates defining the periods of recruitment and follow-up. Report the numbers of individuals at each stage of the study—e.g., Numbers of recruited participants, numbers potentially eligible, examined for eligibility, confirmed eligible, consented, included in the study (screened, sampled), completing follow-up, and analyzed.
Outcomes		Clearly present all characteristics of study participants (e.g., Demographic, source of exposure, covariates) and describe exposures (measures of central tendency and variability).
Numbers analyzed		Number of participants included in each analysis Indicate the number of participants with missing data for each variable of interest. Summarize follow-up time (e.g., average, and total amount), when needed.
Interpretation		For each primary and secondary outcome, results for each group, and the estimated effect size and its precision (such as 95 % confidence interval). For binary outcomes, presentation of both absolute and relative effect sizes is recommended. Report numbers in each exposure category, or summary measures of exposure. Present unadjusted estimates and, if applicable, confounder-adjusted estimates and their precision (e.g., 95 % confidence interval). State the reason for including certain confounders in the models. Comparing the results with HBM guidance values, if exist.
Secondary analyses		Results of any other analyses performed, including subgroup analyses, sensitivity analyses and adjusted analyses, distinguishing pre-specified from exploratory.
**Discussion**		
Key results		Summarize key results with reference to study objectives
Limitations		Limitations, addressing sources of potential bias, imprecision, and, if relevant, multiplicity of analyses
Generalizability		Generalizability (external validity, applicability) of the findings
Interpretation		Give a cautious overall interpretation of results considering objectives, limitations, multiplicity of analyses, results from similar studies, and other relevant evidence.
**Other information**		
Registration		Registration number in www.FAIREHR.com platform, and link to online records
Biobanking	7a	When applicable, provide information regarding biobanking of the biological samples
Funding		Provide the source of funding and the role of the funders for the present study and, if applicable, for the original study on which the present article is based.

## Data Availability

No data was used for the research described in the article.

## Impact Statement

The implementation of the Minimum Information Requirements for Human Biomonitoring (MIR-HBM) is expected to significantly improve the quality, comparability, and reusability of HBM data. By harmonizing data collection, interpretation, and reporting across studies, this guidance enhances cross-study comparability, facilitates regulatory use, and fosters broader collaboration among scientists and policymakers. This initiative will streamline HBM data integration into global databases, promoting more accurate exposure and risk assessments for chemicals, ultimately supporting better-informed decisions for public health and occupational safety.

## Footnotes

1 https://www.wma.net/policies-post/wma-declaration-of-helsinki/.

2 https://www.who.int/news-room/questions-and-answers/item/q-a-ethics-in-public-health-surveillance.

## Appendix A. Supplementary data

Supplementary data to this article can be found online at https://doi.org/10.1016/j.envint.2025.109601.
