# Supplementary material for: Guidance on minimum information requirements (MIR) from designing to reporting human biomonitoring (HBM)
Source: Environ Int. Author manuscript; Available in PMC 2026 Jun 16. (PMC12621227; doi:10.1016/j.envint.2025.109601)
Supplement: Supplement1 [file NIHMS2117004-supplement-Supplement1.docx]

# **Supplementary materials**

# **Annex A: Existing frameworks for human biomonitoring studies**

Over the recent years, international organizations such as the Organization for Economic Co-operation and Development (OECD) (OECD 2022), and the World Health Organization (WHO), APHL - Guidance for laboratory biomonitoring programs (APHL 2019), European Commission - IPCHEM^^[[1]](#footnote-1)^^ data repository for human and environmental monitoring data, along with national authorities, scientific societies like the international Society for Exposure Science (ISES), Society of Environmental Toxicology and Chemistry (SETAC) and communities of practice such as Equator network^^[[2]](#footnote-2)^,^[[3]](#footnote-3)^,^ Elixir toxicology^^[[4]](#footnote-4)^^ and ARRIVE^[[5]](#footnote-5)^ (Du Sert et al. 2020) have developed numerous guidance documents on good practices, reporting standards and tools for different types of scientific data (e.g. in silico, in vitro, in vivo, omics, human data). Using HBM as an exposure assessment approach in Epidemiological studies should follow guidelines and recommendations for ensuring Good Epidemiological Practice (GEP) (Hoffmann et al. 2019; LaKind et al. 2023). The Centers for Disease Control and Prevention’s (CDC’s) National Environmental Public Health Tracking Program (Tracking Program) provides guidance, tools, and examples to help select an appropriate probabilistic sampling design for a population-based biomonitoring study or survey within a state (CDC and Environmental Public Health Tracking Program 2023; Nassif et al. 2021).

Additionally, although peer-reviewed publications consistently report their methods, they often lack sufficient details to allow the full replication of the research or fail to present the information in a standardized, consistent format that would facilitate comparison across studies. However, the situation is improving with the growing use of supplementary materials and the increasing trend of researchers publishing their protocols.

Recommendations on harmonization of HBM studies in Europe emerged mainly via the twin projects COPHES (Consortium to Perform Human Biomonitoring on a European Scale) and DEMOCOPHES (DEMOnstration of a study to Coordinate and Perform Human biomonitoring on a European Scale)(Casteleyn et al. 2015), which led to the Human Biomonitoring for Europe (HBM4EU) project (2017-2022) (Kolossa-Gehring et al. 2023). These HBM activities are now continued in the European Partnership for the Assessment of Risks from Chemicals (PARC) project (Marx-Stoelting et al. 2023). Study protocols and Standard Operating Procedures (SOPs) (Table 1S) were developed within HBM4EU to cover recruitment, informed consent, fieldwork, sampling and result communication among adult general population (Esteban-López et al. 2021; Fiddicke 2021; Mustieles et al. 2020; Pack et al. 2023; Rodríguez-Carrillo et al. 2023; Vorkamp et al. 2021; Zare Jeddi et al. 2021a). Some adaptations would be required to use such protocols in any study designs using HBM approach (Tolonen et al. 2022b). Similar protocols and related SOPs were developed in HBM4EU project for targeted occupational studies focusing on elucidating exposure and early effects in workers in various sectors (Jones et al. 2022; Santonen et al. 2019; Scheepers et al. 2021).

**Annex B**

Examples of available Minimum information requirements in other fields:

1. **Genomics - Minimum Information About a Microarray Experiment (MIAME)**: MIAME standards ensure that microarray data are well-documented and reusable. They require detailed descriptions of the samples used, experimental design, how the arrays were manufactured, how the data were processed, and the normalization methods used.

Reference: Brazma, A., Hingamp, P., Quackenbush, J., Sherlock, G., Spellman, P., Stoeckert, C., Aach, J., Ansorge, W., Ball, C.A., Causton, H.C., Gaasterland, T., Glenisson, P., Holstege, F.C.P., Kim, I.F., Markowitz, V., Matese, J.C., Parkinson, H., Robinson, A., Sarkans, U., Schulze-Kremer, S., Stewart, J., Taylor, R., Vilo, J., Vingron, M. (2001). Minimum information about a microarray experiment (MIAME)—toward standards for microarray data. Nature Genetics, 29(4), 365-371.

1. **Proteomics - Minimum Information About a Proteomics Experiment (MIAPE)**: MIAPE guidelines ensure transparency and reproducibility in proteomics research by detailing the sample preparation methods, the type and settings of the instrumentation used, and the data analysis techniques employed.

Reference: Taylor, C.F., Paton, N.W., Lilley, K.S., Binz, P.-A., Julian, R.K., Jones, A.R., Zhu, W., Apweiler, R., Aebersold, R., Deutsch, E.W., Dunn, M.J., Heck, A.J.R., Leitner, A., Macht, M., Mann, M., Martens, L., Neubert, T.A., Patterson, S.D., Ping, P., Seymour, S.L., Souda, P., Tsugita, A., Vandekerckhove, J., Vondriska, T.M., Whitelegge, J.P., Wilkins, M.R., Xenarios, I., Yates, J.R., Hermjakob, H. (2007). The Minimum Information about a Proteomics Experiment (MIAPE). Nature Biotechnology, 25(8), 887–893.

1. **Environmental Science - Minimum Information About a Simulation Experiment (MIASE)**: MIASE is crucial for ensuring that the results of simulation experiments can be independently verified and reused. Reporting guidelines specifying the information to be provided with the description of a simulation in order to permit its correct interpretation and reproduction.

Reference: Waltemath, D., Adams, R., Bergmann, F.T., Hucka, M., Kolpakov, F., Miller, A.K., Moraru, II, Nickerson, D., Sahle, S., Snoep, J.L., Le Novère, N. (2011). Minimum Information About a Simulation Experiment (MIASE). PLoS Computational Biology, 7(4), e1001122.

1. **Clinical Trials - Consolidated Standards of Reporting Trials (CONSORT)**: CONSORT encompasses various checklists and flow diagrams that guide researchers in reporting randomized controlled trials. They focus on the methodological information necessary to assess the validity and applicability of trial results.

Reference: Schulz, K.F., Altman, D.G., Moher, D., for the CONSORT Group (2010). CONSORT 2010 Statement: updated guidelines for reporting parallel group randomised trials. BMJ, 340, c33

1. **MIRIAM Minimum Information Required in the Annotation of Models**. Reporting guidelines specifying the information to be provided with an encoded model in order to permit its correct interpretation and re-use

Reference: Novère, Nicolas Le; Finney, Andrew; Hucka, Michael; Bhalla, Upinder S; Campagne, Fabien; Collado-Vides, Julio; Crampin, Edmund J; Halstead, Matt; et al. (2005). "Minimum information requested in the annotation of biochemical models (MIRIAM)". Nature Biotechnology. 23 (12): 1509–15. doi:10.1038/nbt1156. hdl:11858/00-001M-0000-0010-853F-C. PMID 16333295.

**6. Metabolomics – Minimum reporting standards** [**for chemical analysis - Metabolomics Standards Initiative (MSI).**](https://pubmed.ncbi.nlm.nih.gov/24039616/) MSI proposes the minimum reporting standards for metabolomics experiments related to the chemical analysis aspects of metabolomics experiments including: sample preparation, experimental analysis, quality control, metabolite identification, and data pre-processing for mass spectrometry and nuclear magnetic resonance spectroscopy. https://github.com/MSI-Metabolomics-Standards-Initiative/CIMR

Reference: Sumner LW, Amberg A, Barrett D, Beale MH, Beger R, Daykin CA, Fan TW, Fiehn O, Goodacre R, Griffin JL, Hankemeier T, Hardy N, Harnly J, Higashi R, Kopka J, Lane AN, Lindon JC, Marriott P, Nicholls AW, Reily MD, Thaden JJ, Viant MR (2007). [Proposed minimum reporting standards for chemical analysis Chemical Analysis Working Group (CAWG) Metabolomics Standards Initiative (MSI).](https://pubmed.ncbi.nlm.nih.gov/24039616/) Metabolomics. 3(3):211-221. doi: 10.1007/s11306-007-0082-2.

Each of these fields has developed specific guidelines to ensure that all necessary information is available to understand, evaluate, and replicate research findings.

**Annex C**

- - - Key elements of **sociodemographic and anthropometric** information include:
  - Age: The individual's age interval, which can affect behavior, health outcomes, and societal roles.
  - Biological Sex at Birth: Refers to the physical and physiological characteristics of an individual. It is determined by anatomy (genitalia, reproductive organs), chromosomes (XX, XY, or variations), and hormones (such as estrogens and testosterone).
  - Gender: The gender identity of the individual, often analyzed to explore differences in behavior, product use (e.g., cosmetics), opportunities, and health outcomes between males, females, and other gender identities.
  - Race and ethnicity: This includes the individual's racial or ethnic background, which can influence cultural practices, social experiences, access to resources, and exposure sources (e.g., ethnic diets) (Flanagin et al. 2021).
  - Education Level: The highest level of education attained, which is often used to assess access to knowledge, job opportunities, and socioeconomic status.
  - Employment Status: Whether the individual is employed, unemployed, retired, or a student, offering insight into economic conditions and social roles.
  - Income Level: The individual's or household's income, providing insight into economic stability, access to services, and quality of life.
  - Adequacy relative to the number of dependents: method to evaluate individual or household income with respect to the number of people it supports to better assess economic well-being and poverty status.
  - Occupation: The type of work or profession an individual is engaged in, indicating potential sources for chemical exposure and access to resources.
  - Marital Status: Whether the individual is single, married, divorced, or widowed, often used to analyze family structure and social support systems.
  - Household Size: The number of people living in the same household, which can impact financial resources, social dynamics, and living conditions.
  - Geographic Location: Where the individual resides (urban, rural, specific regions), which may affect their diet (e.g., country food), access to clean drinking water and air, healthcare, education, and other services.
  - Residential history: record of an individual's places of residence over a specified period.
  - Religion: The individual's religious beliefs, which may influence cultural practices, social behaviors, and values.

Examples of the most common **potential covariates** include:

- - Body Weight and Composition: The ratio of fat to lean tissue influences the storage and processing of chemicals, as lipophilic chemicals tend to accumulate in fat tissues, affecting long-term exposure measurements.
  - Diet and food consumption
  - Personal care products
  - Drug use or medical regimen
  - Alcohol consumption
  - Health Status: Pre-existing health conditions
  - Medical history and medication
  - Occupational history
  - occupational seasonality (full-time, part-time, seasonal), night shifts
  - Lifestyle Factors: Activities like smoking, alcohol consumption, and exercise can influence chemical metabolism and excretion and effects.
  - Environmental Factors: External factors such as temperature, humidity, altitude, age of home or year home built.

**Annex D**

**Table S1**. Commonly used adjustment methods in human biomonitoring

| **Matrix** | **Adjustment Parameter^*^** | **When to use it** | **Conversion Factor** | **Unit for adjusted concentration** | **Consideration** |
| --- | --- | --- | --- | --- | --- |
| Urine | Creatinine concentration | Urinary biomonitoring of non-persistent chemicals to adjust for urine dilutions | Chemical concentration (µg/L) ÷ Creatinine (g/L) | µg/g creatinine | Collect detailed information on the sample collection characteristics including **time of day, time since last void, season of collection, and whether the sample was taken from fasting participants or not and specific activities conducted in the past 24 hours by participants** (e.g. handling of chemicals) (Arbuckle et al. 2014; Ashley-Martin et al. 2023c; Ashley-Martin et al. 2023d; Aylward et al. 2014).  In pregnant women, use specific gravity standardization rather than creatinine correct methods, due to the increased urinary output and creatinine excretion rates (Ashley-Martin et al. 2023a; MacPherson et al. 2018).  The collection of 24h urine samples is not feasible for many study designs or in routine workplace studies, thus, to improve global data comparability, using both creatinine correction and specific gravity adjustments are recommended. |
|  | Specific gravity (SG) adjustment | When hydration may affect urine concentration. Better for cross-population comparisons than creatinine | Chemical concentration × [(SGref-1)/(SG of the sample-1)]  *SGref is often the median specific gravity of the study population. | mg/L or µg/L |  |
| Blood | Lipid content adjustment (Wet Weight to Lipid Weight) | Blood, plasma, serum biomonitoring of lipophilic chemicals | Chemical concentration (µg/L) ÷ total lipid content (g/L) | µg /g lipid | Chemical such as POPs, Dioxins might need lipid concentrations (Canada 2020; O’Brien et al. 2016). |
| Serum/  Plasma | Protein content | For protein-bound chemicals | Chemical concentration (µg/L) ÷ serum or plasma albumin concentration (g/L)  Chemical concentration (µg/L) ÷ serum or plasma creatinine concentration (g/L) | mg/g albumin or ng/g creatinine | Chemicals such as per- and polyfluoroalkyl substances, which are measured in serum and have a high fraction bound to proteins, researchers are encouraged to measure serum albumin or creatinine. This helps account for the potential influence of plasma volume dilution and the resulting pharmacokinetic bias (Andersen et al. 2021; Fischer et al. 2024; Jain and Ducatman 2019). |
|  | plasma volume | For serum or plasma volume changes, especially during pregnancy | Chemical concentration (µg/L) × plasma Volume (L) | mg or µg | If plasma volume is not measured, pregnancy trimester can be used as a proxy. |
| Body | body weight Normalization | Cross-comparison of individuals of different body size (children in comparisons with adults) | Chemical concentration (µg/L) ÷ Body Weight (kg) | µg/kg bw/L | Adjusting the data to a standard body weight. |
|  | Elimination half-life | For persistent chemicals with long half-lives | Chemical concentration (µg/L) at time t= initial concentration of the chemical × $e^{\frac{Time elapsed since exposure}{Elimination half-life of the chemical}}$  ‘e’: Base of the natural logarithm (~2.718) | Remain consistent with the unit of the measured concentration before adjustment (e.g., µg/L) | This adjustment is crucial for estimating peak concentration or exposure dose, especially when collecting samples after a known short-term (acute) exposure. For persistent chemicals, it helps differentiate between ongoing exposure and residual levels due to slow elimination. It is also essential for back-calculating exposure levels if there is a delay between the end of exposure and biomonitoring sample collection, and for comparing populations or time points. |

^*^Please note that for other matrices, such as hair, different normalization approaches may be specified.

**Table S2.** Summary of common human biomonitoring guidance values used for interpretation of biomonitoring data.

| **Guidance values** | **Definition** | **Application^*^** | **Units** | **Study Design considerations** |
| --- | --- | --- | --- | --- |
| Biomonitoring equivalent (BE) | The concentration or range of concentrations of a chemical or its metabolites in a biological medium (blood, urine or other medium) that is consistent with existing health-based exposure guidance values such as a reference dose (RfD) or tolerable daily intake (TDI) (Hays et al. 2008). | General population, used as a screening value to inform chemical prioritization for risk assessment or risk management. It cannot be used to evaluate the likelihood of adverse health effects or for diagnostic purposes. | µg/g creatinine Or  µg/L | Ensure that the biomarker concentration represents quantitative exposure (biomarker adequacy; for example, blood may not be ideal matrix for essential elements due to homeostasis)  The biomonitoring matrix should measure total chemical exposure, including different forms/valency (for e.g., urine antimony concentration does not represent total antimony exposure because only pentavalent antimony predominantly excretes in urine while trivalent excretes in feces).  Studies designed for the general population should have sufficient sample size to address any uncertainties related to inter- and intra-individual variation and kinetic properties of the targeted substance (e.g. limitations associated with chemicals with short elimination half-life in spot urine samples).  Study should be designed to represent the total population of the country or the region.  Should include different age categories, sub-populations of interest (e.g., infants, pregnant women, new immigrants), sex/gender, geographical locations.  Ensure units represent both adjusted and unadjusted concentrations.  Some insight into frequency of exposure and elimination half-life may help to understand whether the concentrations are at steady-state. |
| Human biomonitoring I value HBM-I and Human biomonitoring II value HBM-II | The HBM-I value represents the concentration of a substance in human biological material up to which there is no risk of adverse health effects when considering the individual substance alone. HBM-I values are functionally similar to BE values.  The HBM-II value describes the concentration of a substance in human biological material at and above which adverse health effects are possible and consequently there is an urgent need for exposure reduction and medical advice. For levels between the HBM-I and the HBM-II values adverse health effects cannot be excluded with sufficient certainty, sources of exposure should be identified and minimized or eliminated if this is possible with reasonable efforts (Macey et al. 2025) | General population, used to identify risk for adverse health effects. | µg/L |  |
| Reference Dose (RfD)/Tolerable Daily Intake (TDI) | Derived from toxicological studies; used to calculate biomonitoring levels via reverse dosimetry. | General population- The biomarker concentration should be converted to an external exposure estimate to compare with RfD or TDI (reverse dosimetry). | µg/kg/day |  |
| Human biomonitoring guidance value HBM-GV: HBM-GV_GenPop_  HBM-GV_Workers_ | HBM-GV_GenPop_ represent the concentration of a chemical or its specific metabolite(s) in human biological matrices at which, according to the current state of knowledge, no risk of adverse health effects is to be expected for this individual chemical for a lifetime exposure, and consequently for a single substance approach there is no need for action (Macey et al. 2025). HBM-GV_GenPop_ is identical to BE values and HBM-I values.  The HBM-GVs derived for occupationally exposed adults (HBMGV_Worker_) represent a concentration of a substance or its relevant metabolite(s) in human biological media aiming to protect workers exposed to the respective substance regularly (each work day), and over the course of a working life from the adverse effects related to medium and long-term exposure (Apel et al. 2020) | General population and workers, used to identify risk of health effects anticipated for a lifetime exposure or exposure over the course of working life. | µg/L |  |
| Biological Exposure Indices (BEIs)- American Conference of Governmental Industrial Hygienists (ACGIH) | They represent concentrations of chemicals or their metabolites in biological samples (e.g., blood, urine) that correspond to exposure at the Threshold Limit Value (TLV). focus on toxicological risk assessment | Occupational Biomonitoring | µg/L  µg/g creatinine | Biological matrix and collection timing should align with kinetic properties of the specific chemical (e.g., elimination half-life). |
| Biological Tolerance Values for Occupational Exposures (BATs)- Deutsche Forschungsgemeinschaft (DFG), Germany | BAT values indicate the maximum permissible concentration of substances or metabolites in biological materials of workers exposed to hazardous substances.  Similar to BEIs but focus on toxicological risk assessment. | Occupational Biomonitoring | µg/L |  |
| French Biological Exposure Indices (VLEP-Bs) | VLEP-Bs are similar to BEIs, adapted for France | Occupational Biomonitoring |  |  |

^*^ Human biomonitoring guidance values described here are not recommended to use for individual data interpretation.

**Table S3** examples of the Questionnaires developed under HBM4EU project to capture exposure and confounding variables, available in open access at <https://www.hbm4eu.eu/online-library> .

| **Title** | **Authors** | **Date modified** |
| --- | --- | --- |
| [Overview of HBM4EU harmonised questionnaires and interviewer manuals available in the Online Library](https://www.hbm4eu.eu/online-library/) | Kim Pack, Ulrike Fiddicke | 15-01-2021 11:07 |
| [Substance-specific basic questionnaire (2nd round priority substances: arsenic, benzophenones and mercury) for adolescents (16-19 years) to be applied with support of legal guardians](https://www.hbm4eu.eu/online-library/) | Lead Authors: Marina Lacasaña, Beatriz González-Alzaga and Antonio F Hernández. Full list of authors included in document. | 16-06-2020 14:16 |
| [Substance-specific basic questionnaire (2nd round priority substances: arsenic, benzophenones and mercury) for adolescents (16-19 years) to be applied without support of legal guardians](https://www.hbm4eu.eu/online-library/) | Lead Authors: Marina Lacasaña, Beatriz González-Alzaga and Antonio F Hernández. Full list of authors included in document. | 16-06-2020 14:14 |
| [Substance-specific basic questionnaire (2nd round priority substances: arsenic, benzophenones and mercury) for adolescents (12-15 years)](https://www.hbm4eu.eu/online-library/) | Lead Authors: Marina Lacasaña, Beatriz González-Alzaga and Antonio F Hernández. Full list of authors included in document. | 16-06-2020 14:10 |
| [Substance-specific basic questionnaire (2nd round priority substances: acrylamide, mycotoxins, pesticides and mercury) for children (6-11 years)](https://www.hbm4eu.eu/online-library/) | Lead Authors: Marina Lacasaña, Beatriz González-Alzaga and Antonio F Hernández. Full list of authors included in document. | 16-06-2020 14:07 |
| [Substance-specific basic questionnaire (1st round priority substances) for adolescents (16-19 years) to be applied with support of legal guardians](https://www.hbm4eu.eu/online-library/) | Lead Authors: Marina Lacasaña, Beatriz González-Alzaga and Antonio F Hernández. Full list of authors included in document. | 16-10-2019 15:13 |
| [Substance-specific basic questionnaire (1st round priority substances) for adolescents (16-19 years) to be applied without support of legal guardians](https://www.hbm4eu.eu/online-library/) | Lead Authors: Marina Lacasaña, Beatriz González-Alzaga and Antonio F Hernández. Full list of authors included in document. | 16-10-2019 15:10 |
| [Substance specific basic questionnaire (1st round priority substances) for adolescents (12-15 years)](https://www.hbm4eu.eu/online-library/) | Lead Authors: Marina Lacasaña, Beatriz González-Alzaga and Antonio F Hernández. Full list of authors included in document. | 16-10-2019 15:08 |
| [Substance-specific basic questionnaire (1st round priority substances) for children (6-11 years)](https://www.hbm4eu.eu/online-library/) | Lead Authors: Marina Lacasaña, Beatriz González-Alzaga and Antonio F Hernández. Full list of authors included in document. | 16-10-2019 15:04 |
| [Substance-specific basic questionnaire (2nd round priority substances)](https://www.hbm4eu.eu/online-library/) | Lead Authors: Marina Lacasaña, Beatriz González-Alzaga and Antonio F Hernández. Full list of authors included in document. | 16-10-2019 14:52 |
| [Substance-specific basic questionnaire (1st round priority substances)](https://www.hbm4eu.eu/online-library/) | Lead Authors: Marina Lacasaña, Beatriz González-Alzaga and Antonio F Hernández. Full list of authors included in document. | 16-10-2019 14:49 |
| [Interviewer Manual to the Basic Questionnaire for 2nd round priority substances](https://www.hbm4eu.eu/online-library/) | Marina Lacasaña, Beatriz González-Alzaga and Antonio F Hernández. Full list of authors included in document. | 11-06-2019 14:05 |
| [Matrix-specific questionnaires to accompany the sampling of urine and blood (2nd round priority substances)](https://www.hbm4eu.eu/online-library/) | Marina Lacasaña, Beatriz González-Alzaga and Antonio F Hernández. Full list of authors included in document. | 11-06-2019 14:03 |
| [Interviewer Manual to the basic questionnaire for 1st priority substances](https://www.hbm4eu.eu/online-library/) | Marina Lacasaña, Beatriz González-Alzaga and Antonio F Hernández (EASP). Full list of authors included in the document. | 29-01-2019 15:33 |
| [Concept for the development of non-responder questionnaires in the scope of HBM4EU](https://www.hbm4eu.eu/online-library/) | Kim Pack (UBA), Ulrike Fiddicke (UBA). Full list of authors included in the document. | 29-01-2019 15:28 |
| [Interviewer Manual to the matrix-specific questionnaires (sampling of urine and blood) (1st round priority substances)](https://www.hbm4eu.eu/online-library/) | Kim Pack (UBA). Full list of authors included in the document. | 29-01-2019 15:26 |
| [Matrix-specific questionnaires to accompany the sampling of urine and blood (1st round priority substances)](https://www.hbm4eu.eu/online-library/) | Kim Pack (UBA). Full list of authors included in the document. | 29-01-2019 15:14 |
| [Satisfaction questionnaire](https://www.hbm4eu.eu/online-library/) | Marina Lacasaña and Beatriz González-Alzaga (EASP). Full list of authors included in the document. | 29-01-2019 15:13 |
|  |  |  |
| [Concept for a Study Protocol focussing on Recruitment, Fieldwork](https://www.hbm4eu.eu/online-library/) | Ulrike Fiddicke and Kim Pack (UBA). Full list of authors included in the document. | 13-07-2021 10:37 |
| [Site visit check list and report outline](https://www.hbm4eu.eu/online-library/) | Laura Paalanen (THL), Hanna Tolonen (THL), Anna-Maria Andersson (RegionH), Ulrike Fiddicke (UBA), Loïc Rambaud (ANSP), Romuald Tagne-Fotso (ANSP) | 02-01-2020 11:27 |
| [SOP 1: Selection of Participants and Recruitment](https://www.hbm4eu.eu/online-library/) | Ulrike Fiddicke and Kim Pack (UBA). This document is based on and interconnected with the work done for Deliverable 7.3 and its Annex 1, the Concept for a Study Protocol. Please consider the authors mentioned there. | 29-01-2019 14:17 |
|  |  |  |
| [EN_Chromium(VI)Study_Questionnaire for workplaces (self-administered)](https://www.hbm4eu.eu/online-library/) | Andromachi Katsonouri, Daphne Kleopa and other MOH-CY personnel, Derya Ay and other EEA personnel, Tiina Santonen and the HBM4EU Chromate study working group. | 26-02-2019 10:28 |
| [Occupational Study: Questionnaire for the exposure to hexavalent chromium and other chemicals](https://www.hbm4eu.eu/online-library/) | Sanni Uuksulainen (FIOH), Simo Porras (FIOH) and Karen Galea (IOM). John Cherrie (IOM), Lode Godderis (KU Leuven), Ogier Hanser (INRS), Ivo Iavicoli (DPH), Kate Jones (HSL), Mirja Kiilunen (FIOH), Henriqueta Louro (INSA), Paul Scheepers (RUMC), Henna Veijalainen (FIOH) and Susana Viegas (ESTeSL). | 11-06-2019 14:01 |
| [Occupational Study: Questionnaire for the exposures in E-waste handling](https://www.hbm4eu.eu/online-library/) | Susana Viegas (ESTeSL), Zanna Martinsone (RSU), Linda Matisane (RSU), Karen Galea (IOM), Paul Scheepers (RUMC), Tiina Santonen (FIOH). | 17-06-2020 16:44 |

**Table S4**: Standard Operating Procedures developed under HBM4EU and available on <https://www.hbm4eu.eu/online-library/>

| **Title** | **Authors** | **Date modified** |
| --- | --- | --- |
| [Concept for a Study Protocol focussing on Recruitment, Fieldwork](https://www.hbm4eu.eu/online-library/) | Ulrike Fiddicke and Kim Pack (UBA). Full list of authors included in the document. | 13-07-2021 10:37 |
| [Standard Operation Procedure (SOP) Sample Exchange](https://www.hbm4eu.eu/online-library/) | Dominik Lermen (IBMT), Martina Bartel-Steinbach (IBMT), Frederik Gwinner (IBMT), Sabine Müller (IBMT) Contributors: Agneta Åkesson (KI), Marika Berglund (KI), Anna Bergstrom (KI), Karin Leander (KI), Milena Horvat (IJS), Janja Tratnik (IJS), Argelia Castaño Calvo (ISCIII), Marta Esteban (ISCIII) | 07-02-2020 8:26 |
| [Material and Associated Data Transfer Agreement](https://www.hbm4eu.eu/online-library/) | Dominik Lermen (IBMT), Martina Bartel-Steinbach (IBMT), Frederik Gwinner (IBMT), Sabine Müller (IBMT) Contributors: Agneta Åkesson (KI), Marika Berglund (KI), Anna Bergstrom (KI), Karin Leander (KI), Milena Horvat (IJS), Janja Tratnik (IJS), Argelia Castaño Calvo (ISCIII), Marta Esteban (ISCIII) | 07-02-2020 8:23 |
| [Site visit check list and report outline](https://www.hbm4eu.eu/online-library/) | Laura Paalanen (THL), Hanna Tolonen (THL), Anna-Maria Andersson (RegionH), Ulrike Fiddicke (UBA), Loïc Rambaud (ANSP), Romuald Tagne-Fotso (ANSP) | 02-01-2020 11:27 |
| [Outline of a study protocol for combined HBM and health study](https://www.hbm4eu.eu/online-library/) | Laura Paalanen (THL), Hanna Tolonen (THL), Anna-Maria Andersson (RegionH), Ulrike Fiddicke (UBA), Loïc Rambaud (ANSP), Romuald Tagne-Fotso (ANSP) | 02-01-2020 11:25 |
| [Sample Data Transfer Template](https://www.hbm4eu.eu/online-library/) | Dominik Lermen (IBMT), Martina Bartel-Steinbach (IBMT), Frederik Gwinner (IBMT), Sabine Müller (IBMT) Contributors: Agneta Åkesson (KI), Marika Berglund (KI), Anna Bergstrom (KI), Karin Leander (KI), Milena Horvat (IJS), Janja Tratnik (IJS), Argelia Castaño Calvo (ISCIII), Marta Esteban (ISCIII) | 16-10-2019 15:18 |
| [Corner stone paper for a study on children](https://www.hbm4eu.eu/online-library/) | Ulrike Fiddicke | 11-06-2019 14:06 |
| [HBM4EU-SOP-QA-003 Evaluation of results from Interlaboratory Comparison Investigations (ICI) and External Quality Assurance Schemes (EQUAS)](https://www.hbm4eu.eu/online-library/) | Hans Mol (RIKILT), Thomas Göen (IPASUM), Marta Esteban (ISCIII) | 15-04-2019 10:38 |
| [HBM4EU-SOP-QA-004 Reporting of results of Interlaboratory Comparison Investigations (ICI) and External Quality Assurance Schemes (EQUAS)](https://www.hbm4eu.eu/online-library/) | Hans Mol (RIKILT), Thomas Göen (IPASUM), Marta Esteban (ISCIII) | 15-04-2019 10:38 |
| [HBM4EU-SOP-QA-001 Organisation of Interlaboratory Comparison](https://www.hbm4eu.eu/online-library/) | Hans Mol (RIKILT), Thomas Göen (IPASUM), Marta Esteban (ISCIII) | 15-04-2019 10:37 |
| [HBM4EU-SOP-QA-002 Preparation of control materials for Interlaboratory Comparison Investigations (ICI) and External Quality Assurance Schemes (EQUAS)](https://www.hbm4eu.eu/online-library/) | Hans Mol (RIKILT), Thomas Göen (IPASUM), Marta Esteban (ISCIII) | 15-04-2019 10:37 |
| [SOP 3: Procedure for obtaining human samples](https://www.hbm4eu.eu/online-library/) | Marta Esteban López and Argelia Castaño (ISCIII). Contributors: Susana Pedraza-Diaz (ISCIII), Loïc Rambaud (ANSP) | 29-01-2019 15:11 |
| [SOP 2: Quality Assurance for Recruitment and Fieldwork](https://www.hbm4eu.eu/online-library/) | Ulrike Fiddicke and Kim Pack (UBA). This document is based on and interconnected with the work done for Deliverable 7.3 and its Annex 1, the Concept for a Study Protocol. Please consider the authors mentioned there. | 29-01-2019 14:19 |
| [SOP 1: Selection of Participants and Recruitment](https://www.hbm4eu.eu/online-library/) | Ulrike Fiddicke and Kim Pack (UBA). This document is based on and interconnected with the work done for Deliverable 7.3 and its Annex 1, the Concept for a Study Protocol. Please consider the authors mentioned there. | 29-01-2019 14:17 |
| [Shipping Flowchart](https://www.hbm4eu.eu/online-library/) | Dominik Lermen (IBMT), Martina Bartel-Steinbach (IBMT), Frederik Gwinner (IBMT), Sabine Müller (IBMT) Contributors: Agneta Åkesson (KI), Marika Berglund (KI), Anna Bergstrom (KI), Karin Leander (KI), Milena Horvat (IJS), Janja Tratnik (IJS), Argelia Castaño Calvo (ISCIII), Marta Esteban (ISCIII) | 23-01-2018 14:36 |
| [Sample Transfer Protocol](https://www.hbm4eu.eu/online-library/) | Dominik Lermen (IBMT), Martina Bartel-Steinbach (IBMT), Frederik Gwinner (IBMT), Sabine Müller (IBMT) Contributors: Agneta Åkesson (KI), Marika Berglund (KI), Anna Bergstrom (KI), Karin Leander (KI), Milena Horvat (IJS), Janja Tratnik (IJS), Argelia Castaño Calvo (ISCIII), Marta Esteban (ISCIII) | 23-01-2018 14:33 |
| [Pro-Forma Invoice](https://www.hbm4eu.eu/online-library/) | Dominik Lermen (IBMT), Martina Bartel-Steinbach (IBMT), Frederik Gwinner (IBMT), Sabine Müller (IBMT), Contributors: Agneta Åkesson (KI), Marika Berglund (KI), Anna Bergstrom (KI), Karin Leander (KI), Milena Horvat (IJS), Janja Tratnik (IJS), Argelia Castaño Calvo (ISCIII), Marta Esteban (ISCIII) | 23-01-2018 14:32 |
| [HBM4EU occupational biomonitoring study on hexavalent chromium and other harmful chemicals, Standard Operating Procedures (SOPs)](https://www.hbm4eu.eu/online-library/) | Carina Ladeira, Edna Ribeiro, and Susana Viegas (ESTeSL), Sanni Uuksulainen, Simo Porras, and Tiina Santonen (FIOH), Karen Galea and John Cherrie (IOM), Henriqueta Louro, Célia Ventura, and Maria João Silva (INSA), Elizabeth Leese and Kate Jones (HSL), Ogier Hanser, Sophie Ndaw, and Alain Robert (INRS), Radu-Corneliu Duca, Katrien Poels, and Lode Godderis (KU Leuven), Mirja Kiilunen, Hannu Norppa, Henna Veijalainen, Evgeny Parshintsev, and Tapani Tuomi (FIOH), Flavia Ruggieri and Alessandro Alimonti (ISS), Holger Koch (IPA), Radia Bousoumah, Guillaume Antoine, and Nadège Jacoby (INRS), Darren Musgrove (HSL). | 25-03-2019 14:23 |
| [Occupational Study: Questionnaire for the exposure to hexavalent chromium and other chemicals](https://www.hbm4eu.eu/online-library/) | Sanni Uuksulainen (FIOH), Simo Porras (FIOH) and Karen Galea (IOM). John Cherrie (IOM), Lode Godderis (KU Leuven), Ogier Hanser (INRS), Ivo Iavicoli (DPH), Kate Jones (HSL), Mirja Kiilunen (FIOH), Henriqueta Louro (INSA), Paul Scheepers (RUMC), Henna Veijalainen (FIOH) and Susana Viegas (ESTeSL). | 11-06-2019 14:01 |
| [Occupational Study: Questionnaire for the exposures in E-waste handling](https://www.hbm4eu.eu/online-library/) | Susana Viegas (ESTeSL), Zanna Martinsone (RSU), Linda Matisane (RSU), Karen Galea (IOM), Paul Scheepers (RUMC), Tiina Santonen (FIOH). | 17-06-2020 16:44 |
| [HBM4EU occupational e-waste study: Standard Operating Procedures (SOPs)](https://www.hbm4eu.eu/online-library/) | Lead Author: Tiina Santonen (FIOH). Co-Authors: Susana Viegas (ESTeSL), Ana Maria Tavares (INSA), Henriqueta Louro (INSA), Célia Ventura (INSA), Maria João Silva (INSA), Sophie Ndaw (INRS), Radia Bousoumah (INRS), Kate Jones (HSL), Radu-Corneliu Duca (LNS), Emilie Hardy (LNS), Katrien Poels (KULeuven), Lode Godderis (KULeuven), Karen Galea (IOM), Paul Scheepers (RUMC) | 08-03-2021 15:06 |
| [HBM4EU occupational biomonitoring study on diisocyanates: Standard Operating Procedures (SOPs)](https://www.hbm4eu.eu/online-library/) | Lead Author: Tiina Santonen (FIOH). Co-Authors: Karen Galea (IOM), Bernice Schaddelee-Scholten (TNO), Marika Loikala (FIOH), Radia Bousoumah (INRS), Sophie Ndaw (INRS), Kate Jones (HSL), Elizabeth Leese (HSL), Jade Sumner (HSL), Henriqueta Louro (INSA), Maria João Silva (INSA), Susana Viegas (ESTeSL) | 08-03-2021 15:08 |

1. <https://ipchem.jrc.ec.europa.eu/> [↑](#footnote-ref-1)
2. https://www.equator-network.org/ [↑](#footnote-ref-2)
3. https://www.equator-network.org/library/reporting-guidelines-under-development/reporting-guidelines-under-development-for-observational-studies/ [↑](#footnote-ref-3)
4. https://elixir-europe.org/communities/toxicology [↑](#footnote-ref-4)
5. <https://arriveguidelines.org/> [↑](#footnote-ref-5)
